# Exhibition of Local but Not Systemic Induced Phenolic Defenses in *Vitis vinifera* L. Affected by Brown Wood Streaking, Grapevine Leaf Stripe, and Apoplexy (Esca Complex)

**DOI:** 10.3390/plants8100412

**Published:** 2019-10-14

**Authors:** Piebiep Goufo, Ana C. Marques, Isabel Cortez

**Affiliations:** Centre for the Research and Technology of Agro-Environment and Biological Sciences, Departamento de Agronomia, Universidade de Trás-os-Montes e Alto Douro, Quinta de Prados, 5000-801 Vila Real, Portugal; anamarques@utad.pt (A.C.M.); icortez@utad.pt (I.C.)

**Keywords:** grapevine trunk diseases, signal transduction, systemic acquired resistance, preformed defenses, symptom severity, grapevine trunk diseases

## Abstract

Balance between constitutive and induced responses provides plants flexibility to cope with biotic stresses. This study tested the hypothesis that invasion of grapevine wood by esca-associated fungi induces the production of defensive compounds as part of locally- and systemically-induced responses. In a vineyard, different symptomatic expressions of “Esca complex” in *Vitis vinifera* L. ‘Malvasia’ were evaluated in annual inspections. Then, levels of phenolics and fatty acids were determined in asymptomatic leaves of brown wood streaking (BWS) and grapevine leaf stripe (GLSD) vines, and in symptomatic leaves of GLSD and apoplectic vines; the results were compared with levels in healthy vines. In asymptomatic leaves of BWS and some GLSD vines, levels of phenolics decreased, independent of the total phenolic group. Such responses were usually associated with an increase in levels of linoleic, γ-linolenic and arachidonic acids, well-known signal transduction mediators. In symptomatic leaves, levels of phenolics increased, which is consistent with a locally-induced response; the onset of symptoms coincided with the highest increases e.g., 35% for quercetin-3-*O*-glucuronide. Thus, the long latency period between trunk invasion by fungi and visible foliar damage and the year-to-year fluctuation in symptomatic expressions observed with “Esca complex” might be partially attributed to a better utilization of constitutive defenses.

## 1. Introduction

In their natural environment, plants are at risks of infections by pests and pathogens. Plant resistance to such infections is attributed to multiple defenses that comprise constitutive/preformed and inducible chemical barriers. Inducible chemical barriers involve the synthesis of pathogenesis-related proteins (*PR*) and the accumulation of phytoalexins [1]. An example of a phytoalexin is tricin, which is a flavonoid that confers resistance against brown planthopper in rice [2]. Some phytoalexins are highly species-specific; for example, in pea (*Pisum sativum* L.), the synthesis and localization of pisatin are primarily associated with resistance against Fusarium wilt [3].

“Esca complex” is a widespread and destructive grapevine trunk affliction that affects grape yield and quality [4,5]. The disease is generally associated with the development of diverse wood pathogens among which the ascomycetes *Phaeomoniella chlamydospora* and *Phaeoacremonium minimun* and the basidiomycete *Fomitiporia mediterranea* are most commonly cited [6,7,8,9]. The hyphae of these fungi spread into the xylem vessel and the parenchyma cells of trunks, leading to necrosis [10]. Sometimes, these hyphae invade other woody tissues including cordons, arms, spurs, and canes [11,12,13,14].

“Esca complex” exhibits a long latency time (several years) between wood colonization and visible foliar symptoms [5] and has become increasingly frequent worldwide. A ten-year survey conducted in different vine-growing regions of France revealed that the simplification of the woody vine structure may have resulted in an increase in the incidence of the affliction in the country [15]. Some basic and practical cultural measures for preventing “Esca complex” have been proposed. For example, it was determined that increasing the length of cordons [15] and opting for a minimal pruning system instead of the standard spur-pruning [7] may help minimize the consequences of wood necroses. The foliar application of fertilizer mixtures containing calcium, magnesium, and Fucales seaweed was found effective in reducing foliar symptoms and increasing the yield and quality of berries [4,16]. The correlation between the symptomatic expression of “Esca complex” and the host physiology was highlighted by some authors [13,14,17]. This implies that characterizing the impact of the affliction on grapevine physiology could help in finding candidate biomarkers associated with disease resistance.

Several studies have indicated that phytoalexins and in particular phenolic compounds (phenolic acids, flavonoids, anthocyanins, proanthocyanidins, and stilbenes) play a role in limiting the development of “Esca complex”. A typical reaction to wood colonization by esca-associated fungi is the accumulation of a mixture of polysaccharides (tyloses and gummosis) and the formation of polyphenol-rich reaction zones that obstruct the xylem to compartmentalize the fungi [18]. However, decreased levels of most phenolic compounds were observed in the xylem sap of vines with severe wood symptoms [19], as well as a decreasing trend for the levels of amino acids involved in the biosynthesis of phenolic compounds [20].

Rusjan et al. [10] found that esca-associated fungi caused the accumulation of flavonoids and stilbenes in both asymptomatic and necrotic trunks of vines. In particular, there was a high degree of flavonoid polymerization and a high level of procyanidins in the necrotic wood. However, reduction in the levels of phenolic compounds in asymptomatic wood and no effect on the levels in symptomatic wood were reported for *Pa. chlamydospora*-infected young vines [12]. Further, no difference was observed in total analyzed phenolics in asymptomatic stems of healthy and infected vines in the study by Magnin-Robert et al. [13]; however, a considerable accumulation of stilbenes—*trans*-resveratrol and *trans*-vitisin B—was observed in the affected vines.

It was also demonstrated that vines respond to “Esca complex” by accumulating stilbenes in naturally infected leaves [21] and leaves infected ex vivo with *Pa. chlamydospora* [22]. These increases were accompanied with the up-regulation of phenylalanine ammonia-lyase (*PAL*) and stilbene synthase (*StSy*)—two genes involved in the biosynthesis of polyphenolic compounds—in green [22] and dry leaves [23]. The accumulation of phenolic acids and flavonoids in symptomatic and asymptomatic leaves of field-grown vines was also reported [24]. However, in the study by Martín et al. [25], it was demonstrated that the appearance of foliar symptoms led to a decrease in the levels of flavonoids, proanthocyanidins, and hydroxycinnamic acids in the leaves of *Vitis vinifera* L. ‘Tempranillo’ grown under a dry and warm temperature. For the same cultivar grown under a hot and humid temperature, hydroxycinnamic acids levels increased in symptomatic leaves whereas flavonoids levels decreased. Interestingly, levels of *trans*-resveratrol in asymptomatic leaves of affected vines were slightly higher than those in healthy leaves of non-affected vines in some vineyards in Italy [26].

It is evident from the abovementioned findings that there are different responses of phenolic compounds to “Esca complex”. These different results can be attributed to the types and complexities of symptomatic and asymptomatic materials studied by different authors.

Internal wood symptoms in adult vines are characterized by two diverse shapes of necrotic areas and discolorations. One shape/discoloration is caused by *F. mediterranea* and is called “white rot” or simply “esca” it is characterized by a clear/yellowish soft and spongy mass of wood usually in the center of the trunk or cordons, which can be observed alone or with dark-brown to black spots in the xylem vessels [10,11,14,19]. The second shape/discoloration refers to different types of brown wood necrosis of which “dark/brown wood streaking” (BWS) is most commonly reported; BWS consists of extended columnar strips of xylem necrosis with pink-brown to dark-brown areas or black spots around the annual growth section [13,19]. A third type of shape/discoloration (wood stripe), which is present in external vine wood, is also reported and the symptoms appear as a longitudinal and superficial yellowish-orange stripe and orange-brown discolorations of the young wood vessels located immediately below the bark [27].

Two typical severity levels of leaf symptoms are observed in esca-affected grapevines. A chronic form, characterized by tiger-striped symptoms (GLS) (also named by some authors GLSD for “Grapevine Leaf Stripe Disease”), is initially characterized by chlorosis and then light-green irregular spots and/or scorching between the main veins and/or along the leaf margins. The chlorotic and drying areas gradually expand from the basal to the distal part of the leaves, and then they coalesce to become partial necrotic stripes. As the chlorotic tissues turn yellow-brown or red-brown, the leaves exhibit a tiger stripe pattern [17,19,25,26,27,28]. GLSD symptoms are also reported in the berries and consist of tiny dark-brown or purple speckling distributed irregularly over the entire surface or scattered at the far end (termed “black measles” by some authors) and sometimes of shriveling/withering of grape bunches [18]. “Apoplexy” consists of partial or complete sudden wilting of the crown and is considered an acute form of the leaf symptomatic expression of “Esca complex” [23,27] or an acute form of GLSD by some authors [14]. BWS and GLSD vines are associated with a large procession of inhabiting fungi, although *Pa. chlamydospora* and *Pm. minimun* are most commonly found [18]. Although the percentage of necrotic areas within the wood from which pathogens can be isolated is often a key factor to determine the severity of ”Esca complex,” wood necrosis is not always related to the incidence of foliar symptoms [15]. Given this observation, the term “esca proper” is used by some authors to indicate the coexistence of “white rot” and GLSD in the same vine [13,14]. “Esca complex” is most commonly noted in established vineyards. In newly planted vines, scattered brown-black spots of necrotic xylem (without decay), often with a dark viscous ooze and a moderate/diffuse chlorosis of the leaves are observed, and the disease is termed “Petri disease” [6,8,12].

Studies have shown that many abiotic factors and cultural practices (alone or combined) may influence the development of “Esca complex” and the variability of its damage [15,20,26]. For example, it has been observed that heavy rainfall followed by hot winds in mid-summer favors the onset of apoplexy [18]. These observations were confirmed by other authors [25], who then reported that the biosynthesis of phenolic compounds in esca-affected leaves depended on the climate under which the vines were grown.

The above literature review shows that potential defense mechanisms developed by grapevine to resist esca-associated fungi need to be explored further. Therefore, the experiment in this study was designed to produce complementary data that would help improve the understanding of defense events occurring during an “Esca” invasion. It was hypothesized that esca-associated fungi induce the production of defensive compounds in leaves as part of both a locally- and systemically-induced defense response; local induction is defined as the enhancement of defensive traits in the organ that is attacked, while systemic induction is the enhancement of defenses in distant and undamaged organs, conferring broad-spectrum resistance throughout the plant [29,30]. To test this hypothesis, the accumulation of polyphenols in the leaves of vineyard-grown plants was monitored and levels of fatty acids were determined. Recent studies demonstrated that fatty acids play an important role in the modulation of signal transduction pathways in systemically acquired pathogen resistance. In several plants, the degree of resistance to pathogens was found to be directly correlated with the levels of C16:1 (palmitoleic acid), C18:1 (oleic and elaidic acids), C18:2 (linoleic and linolelaidic acids), C18:3 (α-linolenic and γ-linolenic acids), and C20:4 (achidonic acid) [1]. For example, rhizobacteria-induced enhanced resistance to *Botrytis cinerea* is associated with the accumulation of C18:2 and C18:3 in *Phaseolus vulgaris* L. [31], while reduction in C18:1 level induces defense responses against several pathogens by upregulating expressions of a variety of structurally diverse *R* genes in *Arabidopsis* [32]. Therefore, the levels of phenolic compounds and fatty acids in asymptomatic and symptomatic leaves of grapevine affected by BWS, GLSD and apoplexy were investigated to identify infection stages at which plant resistance mechanisms were more efficiently activated.

## 2. Results

### 2.1. Effect of Brown Wood Streaking, Grapevine Leaf Stripe and Apoplexy on the Levels of Phenolic Compounds in Grapevine Leaves

**Figure 1:**
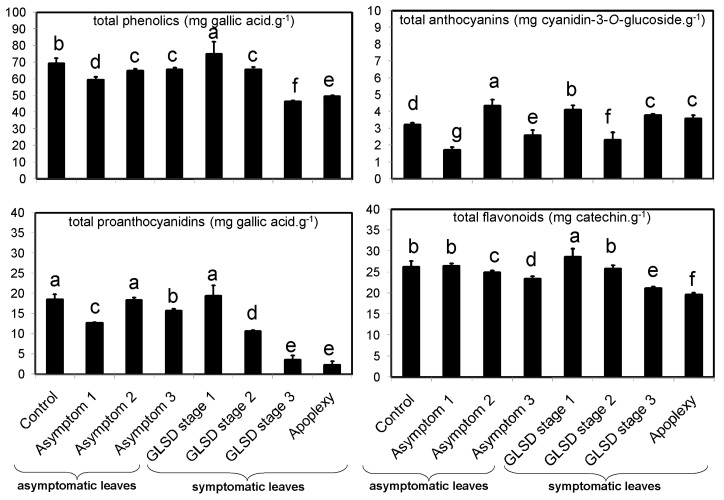
Total phenolic (TPC), anthocyanin (TAC), proanthocyanidin (TPAC), and flavonoid (TFC) content (dry weight basis) in asymptomatic and symptomatic leaves of vines affected by brown wood streaking, grapevine leaf stripe and apoplexy. The legend is as in Figure 6. Error bars = standard deviations (*n* = 4); different letters above the columns denote statistical differences (Tukey’s test; *P* ≤ 0.05).

In this study, the amount of total phenolic compounds in the leaves of vines affected by “Esca complex” was first analyzed using colorimetric methods (Figure 1). An interesting trend emerged in that asymptomatic leaves of BWS and GLSD vines had a lower amount of TPC than that in control leaves, with a decrease of 14% in leaves of BWS vines (asymptomatic 1). The amount of TPC was particularly high in leaves exhibiting the initial foliar symptoms (GLSD stage 1) as compared to that in control leaves, and then, it decreased in proportion to the severity of chlorosis and necrosis on the leaves. Changes in the amount of TAC, TPAC, and TFC due to BWS and GLSD were similar to changes in the amount of TPC, with some exceptions: the highest amount of TAC was measured in asymptomatic leaves of GLSD vines that had both symptomatic and asymptomatic cordons (asymptomatic 2), and a 64% increase in the amount of TAC was recorded passing from chlorotic/spotting/scorching leaves (GLSD stage 2) to tiger striped (GLSD stage 3) and apoplectic leaves (Figure 1).

The HPLC method used in this study led to the separation of 104 peaks with 95 peaks showing phenolic characteristics. Using the information provided by the detector and reports in the literature, the peaks were assigned to metabolites of the structure classes hydroxybenzoic acid (9), hydroxydiphenic acid (1), proanthocyanidin (9), stilbene (1), hydroxycinnamic acid (16), flavonoid (37), and anthocyanin (10). Eight metabolites were labeled “unknown,” whereas four exhibited the characteristics of both proanthocyanidins and hydroxybenzoic acids and were labeled as “benzoic acid derivatives.” On average, quercetin-3-*O*-glucuronide was the major phenolic compound in the leaves (2834.43 mg·kg^−1^) followed by myricetin-3-*O*-galactoside (127.68 mg·kg^−1^), quercetin-3-*O*-glucoside (127.32 mg·kg^−1^), quercetin-3-*O*-galactoside (83.35 mg·kg^−1^), caftaric acid (82.78 mg·kg^−1^), myricetin-3-*O*-glucoside (60.82 mg·kg^−1^), kaempferol-3-*O*-glucoside (54.01 mg·kg^−1^), coutaric acid (49.00 mg·kg^−1^), epicatechin (37.93 mg·kg^−1^), quercetin-3-*O*-rutinoside (23.10 mg·kg^−1^), kaempferol-3-*O*-rutinoside (13.72 mg·kg^−1^), and epigallocatechin gallate (12.38 mg·kg^−1^). The levels of the remaining compounds were below 10 mg·kg^−1^ (Table S1). In some samples, quercetin-3-*O*-glucuronide and quercetin-3-*O*-glucoside co-eluted in the chromatograms; therefore, the levels of these two compounds were summed and used in the statistical analyses; the same was true for myricetin-3-*O*-galactoside and myricetin-3-*O*-glucuronide. In *V. vinifera,* several stilbenes have been reported as stress response metabolites [22]. In this study, only one stilbene was detected, which was identified as *trans*-resveratrol, with an average content of 0.98 mg·kg^−1^ (Table S1). This value was substantially lower than 1.38–50.49 mg·kg^−1^, which was observed in the leaves of some Italian cultivars [26]. The non-detection of stilbenes was not surprising because stilbenic compounds are usually detected by HPLC from a filtrate obtained after several solid–liquid and liquid–liquid extraction and purification steps [33]. The clean-up step used in this study was aimed at discarding chlorophylls and chromatography was optimized for the separation of flavonoids and proanthocyanidins.

The major compounds (average content ≥ 1.00 mg·kg^−1^; Table S1) were first analyzed using ANOVA. This analysis allowed two main categories of compounds to be delineated, on the basis of similar trends in the contents observed comparing control, asymptomatic and symptomatic leaves.

**Figure 2:**
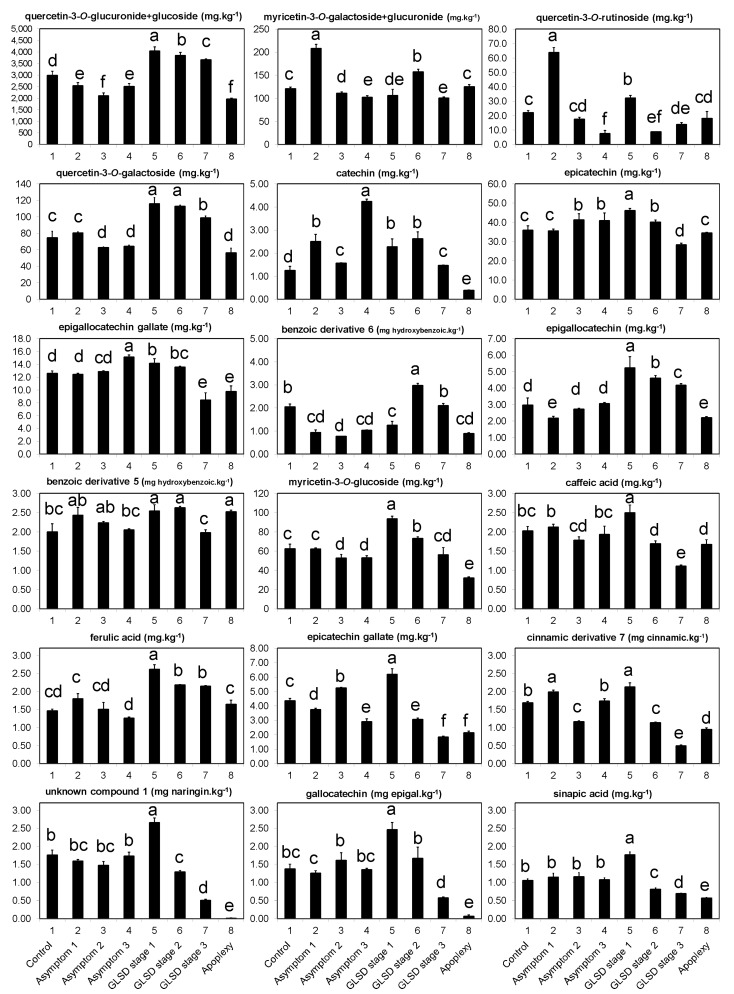
Phenolic compounds in asymptomatic and symptomatic leaves of vines affected by brown wood streaking, grapevine leaf stripe and apoplexy; their levels (dry weight basis) progressively decreased with increasing severity of symptoms. The legend is as in Figure 6. Error bars = standard deviations (*n* = 4); different letters above the columns denote statistical differences (Tukey’s test; *P* ≤ 0.05); hydroxybenzoic = hydroxybenzoic acid; cinnamic = cinnamic acid; and epigal = epigallocatechin.

The first category (Figure 2) consisted of 20 compounds that showed three characteristics. (i) The levels of these compounds were particularly high in symptomatic leaves exhibiting the initial foliar symptoms of GLSD (GLSD stage 1) compared with those in control and asymptomatic leaves. The percentage increase between control and GLSD stage 1 leaves ranged from 13% (epigallocatechin gallate) to 81% (catechin). However, there were some exceptions: the levels of myricetin-3-*O*-galactoside+myricetin-3-*O*-glucuronide and quercetin-3-*O*-rutinoside were the highest in asymptomatic leaves of BWS vines (asymptomatic 1), and the levels of epigallocatechin gallate and catechin were the highest in asymptomatic leaves of GLSD vines with berry symptoms (asymptomatic 3). (ii) The levels of these compounds progressively decreased with the increasing severity of the leaf symptom, with the lowest values usually being measured in apoplectic leaves. However, for some compounds a slight level increase was observed in apoplectic leaves as compared to tiger striped leaves (GLSD stage 3); this suggests that apoplexy might not only be a severe form of GLSD. These compounds included epicatechin, benzoic acid derivative 5, caffeic acid, hydroxycinnamic acid derivative 7, myricetin-3-*O*-galactoside+myricetin-3-*O*-glucuronide, and quercetin-3-*O*-rutinoside. (iii) The levels of these compounds were generally similar in asymptomatic leaves or lower in asymptomatic leaves of BWS and GLSD vines than those in control leaves. For example, the levels of quercetin-3-*O*-glucuronide+quercetin-3-*O*-glucoside decreased by 14, 29, and 16% in asymptomatic leaves of BWS (asymptomatic 1), GLSD foliar-symptomatic (asymptomatic 2), and GLSD berry-symptomatic (asymptomatic 3) vines, respectively. The levels of only a few compounds increased in asymptomatic leaves and that included an 18, 71, 188, 70, 99, 20, and 17 increase for hydroxycinnamic acid derivative 7, myricetin-3-*O*-galactoside+myricetin-3-*O*-glucuronide, quercetin-3-*O*-rutinoside in asymptomatic leaves of BWS vines, catechin in asymptomatic leaves of BWS vines, catechin in asymptomatic leaves of GLSD berry-symptomatic vines, epicatechin gallate in asymptomatic leaves of GLSD foliar-symptomatic vines, and epigallocatechin gallate in asymptomatic leaves of GLSD berry-symptomatic vines, respectively.

**Figure 3:**
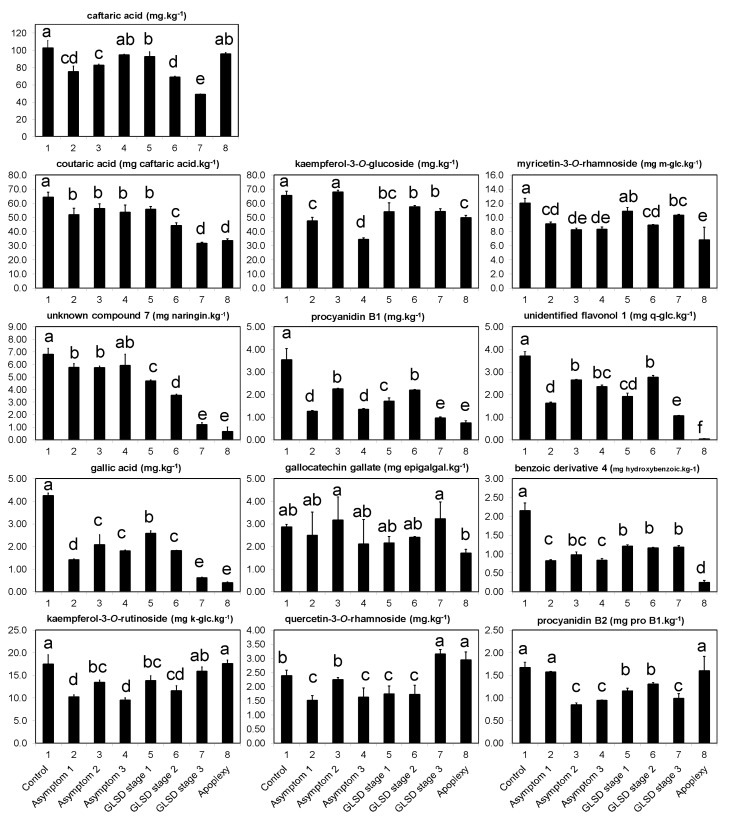
Phenolic compounds in asymptomatic and symptomatic leaves of vines affected by brown wood streaking, grapevine leaf stripe and apoplexy, with the highest levels (dry weight basis) in control leaves. The legend is as in Figure 6. Error bars = standard deviations (*n* = 4); different letters above the columns denote statistical differences (Tukey’s test; *P* ≤ 0.05); k-glc = kaempferol-3-*O*-glucoside; m-glc = myricetin-3-*O*-glucoside; epigalgal = epigallocatechin gallate; q-glc = quercetin-3-*O*-glucoside; hydroxybenzoic = hydroxybenzoic acid; and pro B1 = procyanidin B1.

The second category (Figure 3) consisted of 13 compounds whose levels were usually the highest in control leaves, confirming the general trend of decreased phenolic content in asymptomatic leaves of BWS and GLSD vines as observed in Figure 1; Figure 2. Caftaric acid—the main hydroxycinnamic acid identified in this study—belonged to that category and its levels decreased by 27% (*P* ≤ 0.05), 20% (*P* ≤ 0.05), and 8% (*P* > 0.05) in asymptomatic leaves of BWS, GLSD foliar-symptomatic, and GLSD berry-symptomatic vines, respectively. The levels of these compounds usually decreased in proportion to the severity of the chlorosis and necrosis on symptomatic leaves, as observed clearly for unknown compound 7 and coutaric acid. Few exceptions were kaempferol-3-*O*-rutinoside and quercetin-3-*O*-rhamnoside, whose levels tended to increase with increasing severity of symptoms.

**Figure 4:**
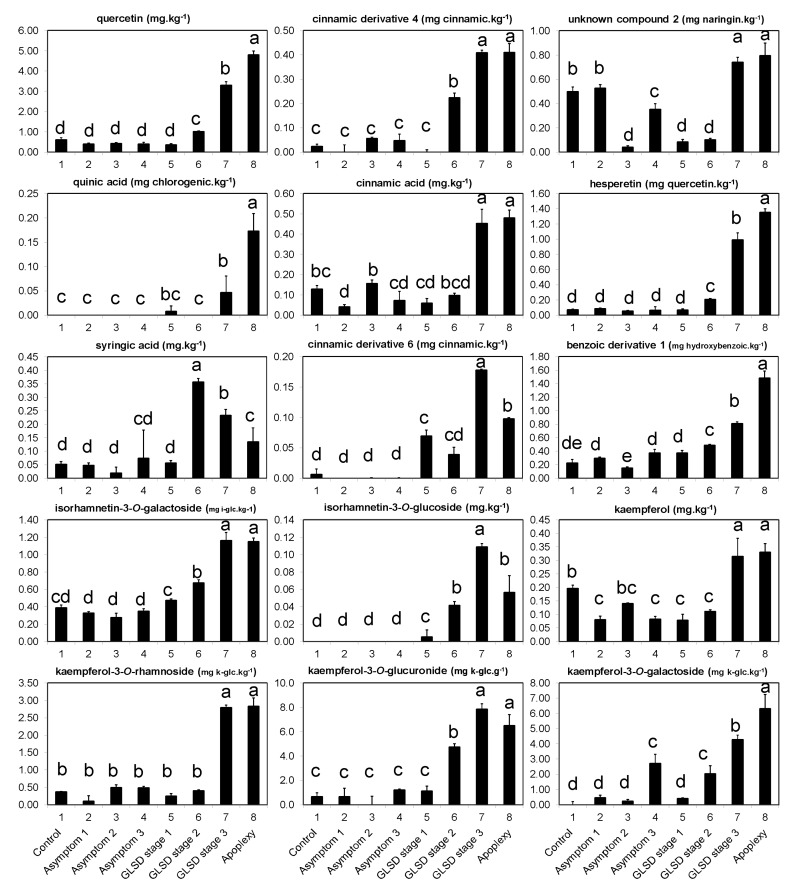
Phenolic compounds in asymptomatic and symptomatic leaves of vines affected by brown wood streaking, grapevine leaf stripe and apoplexy; their levels (dry weight basis) progressively increased with increasing severity of symptoms. The legend is as in Figure 6. Error bars = standard deviations (*n* = 4); different letters above the columns denote statistical differences (Tukey’s test; *P* ≤ 0.05); cinnamic = cinnamic acid; chlorogenic = chlorogenic acid; hydroxybenzoic = hydroxybenzoic acid; i-glc = isorhamnetin-3-*O*-glucoside; and k-glc = kaempferol-3-*O*-glucoside.

A principal component analysis (PCA) was also performed to identify additional minor compounds that could help further classify the different leaf-groups. On the loading plot, 15 compounds were clearly separated from the other compounds (Figure S1). Interestingly, the levels of these compounds progressively increased with increasing symptom severity, showing strong correlations (Table S1), and reaching on average a 704% increase between control and apoplectic leaves (Figure 4). These compounds were usually undetected or detected at very low levels in asymptomatic leaves. Moreover, with the exception of quercetin, kaempferol-3-*O*-glucuronide, and kaempferol-3-*O*-galactoside, all these other compounds had average contents < 1 mg·kg^−1^. In particular, GLSD and apoplexy stimulated the production of quinic acid, hydroxycinnamic derivative 6, and isorhamnetin-3-*O*-glucoside, which were not detected in most asymptomatic leaves.

Overall, the levels of the remaining minor compounds were not affected in asymptomatic leaves, with the exception of four compounds that were detected primarily in these leaves (hydroxycinnamic acid derivative 2, unknown compound 4, unidentified flavonol 8, and *p*-hydroxybenzoic acid). For symptomatic leaves, the levels of some remaining minor compounds increased, while those of the others decreased with the increasing severity of symptoms (Table S2).

### 2.2. Effect of Brown Wood Streaking, Grapevine Leaf Stripe and Apoplexy on the Levels of Fatty Acids in Grapevine Leaves

In this study, 35 fatty acids present in grapevine leaves were separated by GC: 15 saturated fatty acids (SFA), 9 monounsaturated fatty acids (MUFA), and 11 polyunsaturated fatty acids (PUFA). The predominant fatty acids were γ-linolenic (C18:3n6; 34.65%), palmitic (C16:0; 15.09%), linoleic (C18:2n6c; 5.81%), elaidic (C18:1n9t; 5.49%), palmitoleic (C16:1n7; 3.71%), α-linolenic (C18:3n3; 3.35%), cis-4,7,10,13,16,19-docosahexaenoic (C22:6n3; 3.14%), caprylic (C8:0; 3.05%), arachidic (C20:0; 2.81%), and oleic (C18:1n9c; 2.22%) acids (Table S3).

**Figure 5:**
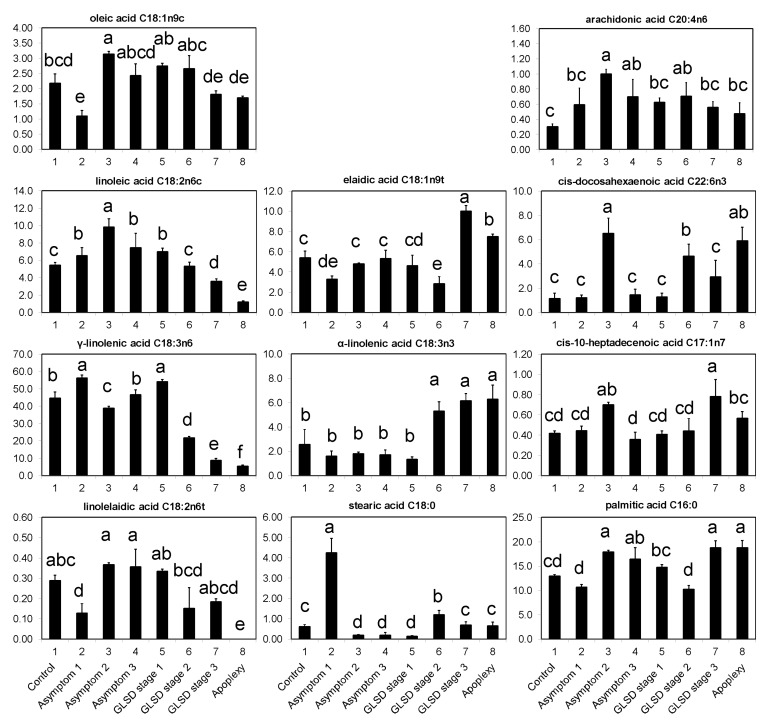
Fatty acids in asymptomatic and symptomatic leaves of vines affected by brown wood streaking, grapevine leaf stripe and apoplexy; their levels (%) were usually higher in asymptomatic leaves than in control leaves. The legend is as in Figure 6. Error bars = standard deviations (*n* = 4); different letters above the columns denote statistical differences (Tukey’s test; *P* ≤ 0.05).

The levels of most fatty acids were lower in asymptomatic leaves of BWS and GLSD vines than in control leaves (Table S4). Interestingly, the majority of C18 compounds and some other fatty acids did not seem to exhibit this decreased pattern (Figure S2; Figure 5).

In general, asymptomatic leaves of BWS vines (asymptomatic 1) had lower levels of C18:1n9c, C18:1n9t, C18:2n6t (linolelaidic acid) (*P* ≤ 0.05), and C18:3n3 (*P* > 0.05) than control leaves; however, the levels of C18:2n6c, C18:0 (stearic acid), and C18:3n6 were higher in asymptomatic leaves of BWS vines than in control leaves. In particular, a 600% increase was observed for C18:0. Compared to control leaves, asymptomatic leaves of GLSD vines with both asymptomatic and symptomatic cordons (asymptomatic 2) had higher levels of C18:1n9c, C18:2n6c, and C18:2n6t, and lower levels of C18:0 and C18:3n6, while no change was recorded for C18:1n9t and C18:3n3. C18 levels in asymptomatic leaves of GLSD berry-symptomatic vines (asymptomatic 3) responded similarly to “Esca” attack as those in asymptomatic leaves of GLSD foliar-symptomatic vines, with the exception of C18:3n6, whose level remained unchanged. Substantial differences between control and asymptomatic leaves were also observed with regards to the levels of C16:0, C17:1n7 (cis-10-heptadecenoic acid), C20:4n6 (arachidonic acid), and C22:6n3. In all asymptomatic leaves, there was a strong increase in C20:4n6 levels. The levels of C17:1n7, C22:6n3, and C16:0 increased in asymptomatic leaves of GLSD foliar-symptomatic vines, while the level of C16:0 increased in asymptomatic leaves of GLSD berry-symptomatic vines (Figure 5).

In symptomatic leaves, a distinct correlation between disease symptom severity and fatty acid levels was observed (Table S3). Overall, leaf symptom severity was positively correlated with the levels of SFA (with the exception of tricosanoic acid C23:0 and heptadecanoic acid C17:0), MUFA (with the exception of C18:1n9c), and n3-PUFA (with the exception of cis-5,8,11,14,17-eicosapentaenoic acid C20:5n3), and negatively correlated with the levels of n6-PUFA (with the exception of cis-11,14-eicosadienoic acid C20:2n6) (Figure 5; Tables S3 and S4).

## 3. Discussion

In this study, great variability was observed in the accumulation of phenolic compounds and fatty acids in grapevine as a response to infection by esca-associated fungi, which indicated that dynamic and transient metabolic changes occur when symptoms spread from the trunk to the leaves.

### 3.1. Exhibition of Locally Induced Defenses in Symptomatic Leaves

It was clear from the data in Figure 1 and Figure 2 that the levels of phenolic compounds increased in symptomatic leaves of GLSD vines exhibiting the first symptoms of the disease. The precocity of pathogen recognition and the velocity of the activation of defense responses are keys to enhancing the resistance of plants to infections [1,22,34]. The recognition of esca-related pathogens by grapevine plants and the formation of foliar symptoms are debated topics because propagules of *Pa. chlamydospora*, *Pm. minimun*, and *F. mediterranea* have never been detected on the leaves [18]. The most accepted interpretation is that toxic metabolites secreted by esca-associated fungi or resulting from reaction products of the infected wood are translocated from the xylem to the leaves via the transpiration/sap stream, which thus incites foliar symptom development [18]. This assumption suggests that the fungi induced local defense responses in grapevine when their metabolites reached the host leaf cells. In fact, foliar administration of calcium and subsequent accumulation of calmodulin, that mitigate the effect of the plant response, reduced GLSD leaf symptom expression [16]. The increase in the levels of phenolic compounds was the greatest when GLSD symptoms started appearing on the leaves. However, with increasing symptom severity, the levels of these compounds decreased. It is reported that the resistance of plants to infections depends partly on the balance between production/degradation of defensive compounds [19]. *Phaeomoniella chlamydospora* and *Pm. minimun* produce several enzymes that are known to travel in the plant and could reach the leaves [19]. However, the hypothesis of a phenolic decrease caused by enzymatic activities of esca-associated fungi is not tenable because these fungi lack enzymes such as ligninases, which would enable them to degrade specific phenolic bonds [9]. These decreases were also unlikely to be caused merely by chlorosis and necrosis. It is known that the development of GLSD necrotic areas in leaves leads to a decrease in photosynthetic assimilation [23]. Similarly, the expression of photosynthesis-related genes is strongly repressed in apoplectic leaves [28]. However, the reduced photosynthesis did coincide with the accumulation of hexoses and phenolic compounds in the studies by [28] and [25], respectively. It is conceivable that vines with reduced photosynthetic activity have to face with high levels of reactive oxygen species (ROS) and this can compromise the biosynthesis of primary and secondary metabolites [17,23]; this would suggest that, although the leaves initially respond to the infection with an increased production of phenolics, the vines no longer have the resources to support secondary metabolite production with increasing symptom severity, at least for many compounds detected in this study. Lambert et al. [22] also observed that the levels of *trans*-piceid and *trans*-resveratrol began to increase in grapevine leaves after 3 h of post treatment with a *Pa. chlamydospora* culture filtrate, and then decreased at 24 h. In grapevine leaf disks artificially infected with *Erysiphe necator*—a causal agent of grapevine powdery mildew—the levels of stilbenes also increased 1 to 3 days post inoculation, and then decreased with increasing disease symptom severity [34].

Interestingly, it was found that the levels of several compounds increased with increasing disease symptom severity (Figure 4). The majority of these compounds were undetected or at very low levels in asymptomatic leaves. The term “phytoalexin” has been used to describe compounds that are absent or normally present at low levels in cells, but which may increase enormously after infection and specifically inhibit the growth of a parasite [31,34]. In grapevine, the principal stress response phytoalexins studied are stilbenes [19,33]. In the study by Calzarano et al. [26], the time course of four stilbenes (*trans*-resveratrol, *trans*-ε-viniferin, *trans*-δ-viniferin, and *trans*-pterostilbene) was examined in vine leaves with different degrees of GLSD symptoms; in general, phytoalexin levels increased with increasing leaf symptom severity, although that depended on the growth stage of the plants. In this study, only *trans*-resveratrol was detected in the leaves; however, it was detected at very low amounts because of the extraction method adopted, which did not allow a clear assessment of the impact of the infection (Table S2). In a subsequent study, BWS, GLSD stage 1 and GLSD stage 2 samples were submitted to a metabolomic analysis, and ca., six stilbenes were identified in the leaves; levels of *trans*-piceid, *trans-*ε-viniferin, Ampelopsin A and *trans*-Pterostilbene increased with increasing leaf symptom severity, confirming the report in [26]. The level of a resveratrol dimer decreased, while the level of *trans*-resveratrol remained unchanged (*personal communication*). Compounds detected in Figure 4 could also act as phytoalexin in *Vitis vinifera* ‘Malvasia’. The net accumulation of these compounds and stilbenes within the infected leaves may contribute to grapevine’s ability to minimize the spread of the disease. A parallel could be drawn with the case of powdery mildew described by [34], where the de novo synthesis of ε-viniferin and δ-viniferin at the site of infection coincided with the interruption of the pathogen cycle. The induction of several defense genes and proteins following “Esca attack” has been reported by some authors, which strengthen the finding of this study. For example, in the study by Letousey et al. [23], the expression of the defense-related genes *StSy* (stilbene synthase), *PAL* (phenylalanine ammonialyase), *Chi4C* (class IV chitinase), *Chit1b* (class I basic chitinase), and *GST1* (glutathione-*S*-transferase) was strongly upregulated in dried leaves of apoplectic vines, whereas *SOD* (superoxide dismutase) was repressed. Similar inductions of genes encoding chitinases, stilbenic phytoalexins, and *PR* proteins (*Chit1b*; *CHV5*, *STS*, *GST5, SOD,* and *PR-6*) were observed in GLSD leaves [25]. The expression levels of PR-5 proteins, POX (peroxidase), and catechol PPO (polyphenol oxidase) were also higher in symptomatic and asymptomatic stems of grapevine affected with esca proper and apoplexy, than in the healthy vines [14].

Overall, leaf symptom severity was positively correlated with SFA, MUFA, and n3-PUFA levels, and negatively correlated with n6-PUFA levels (Figure 5; Table S4). The increased levels of SFA and MUFA observed in tiger striped and apoplectic leaves might be beneficial as they provide energy for various metabolic processes, which is particularly important for the energy-intensive processes that underlie the plant defense response. In particular, C16 and C18 fatty acids are important precursors of cuticular wax synthesis [1]. Thus, they strengthen cell membranes, provide structural integrity, and hamper the infiltration and spread of pathogens into the leaves.

### 3.2. Absence of Systemically Induced Defenses in Asymptomatic Leaves

Data in Figure 1 and Figure 3 shows that, before the appearance of foliar symptoms, the presence of esca-associated fungi in the wood caused a decrease in the levels of phenolic compounds in the leaves. Only a few systemic responses for six compounds were recorded in asymptomatic leaves, including a 71% and 188% increase for myricetin-3-*O*-galactoside+myricetin-3-*O*-glucuronide and quercetin-3-*O*-rutinoside in asymptomatic leaves of BWS vines, respectively (Figure 2). These results do not support the hypothesis of a systemic induction of phenolic compounds in grapevine leaves; this was an unexpected finding given the slight increase in stilbenes in asymptomatic leaves of *V. vinifera* L. ‘Trebbiano d’Abruzzo’ [26] and in flavonoids and phenolic acids in asymptomatic leaves of *V. vinifera* L. ‘Alvarinho’ [24]. However, the findings of this study agree with some previous reports; Magnin-Robert et al. [17] found that the expression of the defense-related genes *GLUC* (β-1,3-glucanase), *GTS1*, *StSy, CHV5,* and *PAL* were repressed in most pre-GLSD leaves, while *Chit1b* and *Chi4C* were not affected, although the expression of stress-related genes was stimulated in vines with a decrease of net photosynthesis >75%. In most studies, *SOD* expression was found to decrease or showed a decreasing trend in both pre-apoplectic and pre-GLSD leaves [13,17,23]. In asymptomatic wood of apoplectic and esca proper vines, several genes and proteins involved in phenylpropanoid metabolism were either down- or upregulated [13,14] e.g., *IFRhom* and *IFRL4* (isoflavone reductase) and *leucoAND* (leucoanthocyanidin dioxygenase). In contrast, Valtaud et al. [28] observed an enhancement of mRNAs encoding *PR-10*, *Chi1b*, and *Chi3* (endochitinase 3) genes in asymptomatic leaves of GLSD vines with both symptomatic and asymptomatic cordons similar to those studied in this study. Letousey et al. [23] also reported an induction of *PAL, StSy, Chi4C*, *Chit1b*, and *GST1* genes in pre-apoplectic leaves.

The decline in the levels of phenolic compounds in asymptomatic leaves of BWS and GLSD vines was concomitant with the accumulation of C18:2n6c, C18:3n6, and C20:4n6, and generally a decrease in C18:1n9c levels in the leaves of BWS vines (Figure 5). These fatty acids are most prominently known for their specific signaling roles in plant defenses and they regulate ROS and nitric oxide (NO) levels by inducing specific effects on ROS- and NO-generating enzymes. For example, C18:1 in low amounts physically associates with the chloroplastic NITRIC OXIDE ASSOCIATED1 (*NOA1*) protein, inhibiting its GTPase and promoting its proteolytic turnover, which generates NO, triggering the transcriptional upregulation of NO-responsive nuclear genes, and thereby activating disease resistance [1,31,32]. Fatty acid data from this study show that systemic changes in fatty acid flux also occurred in the distal organs of grapevine. This would suggest that a mobile signal at the site of local infection is translocated to the leaves. Yet, information on the effector molecules involved in long-distance defense signaling in plants remains lacking. In tomato and other Solanaceous plants, systemic signaling appears to be mediated by systemin, an 18-amino acid peptide. Systemin is produced by wounded leaf cells, and travels to companion cells where it binds to a receptor, triggering the accumulation of jasmonic acid and fatty acids [29]. *Phaeomoniella chlamydospora, Pm. minimun*, and *F. mediterranea*, the major esca-wood-infesting fungi, were shown to produce diverse toxic metabolites detectable in the leaves. *Phaeomoniella chlamydospora* and *Pm. minimun* produce scytalone, 4-hydroxyscytalone, isosclerone [13,19,35], and pullulan [19], among other compounds. In the case of *F. mediterranea,* metabolites secreted that can be considered as toxic include 4-hydroxybenzaldehyde, dihydroactinolide, and 6-methoxymellein [35]. These toxic compounds have been identified in higher amounts in symptomatic than in asymptomatic leaves of affected vines [19]. Further, they may function as both pathogenic and virulence factors, thus representing the specific signals sensed by grapevine distal organs that result in fatty acid accumulation in asymptomatic leaves.

Specific changes in the levels of these fatty acids indicate that asymptomatic leaves are mounting a defense response in time to cope with the infection. That assumption is in good agreement with previous studies that report biochemical and physiological changes in grapevine leaves before the appearance of visible symptoms. For example, downregulation of *SOD* [23], low abundance of the *SODCP* protein *s6205* [14], upregulation of *GST1* [23], high abundance of GSTU1 and GSTF2 proteins, enhanced activity of *GST5* [28], and a decrease in the number and size of starch grains [28] have been reported in asymptomatic leaves of esca-affected grapevine as an early response of cells distant from the damaged wood. Within the week preceding leaf symptoms, drastic physiological alterations of photosynthesis were also registered in pre-apoplectic and pre-GLSD leaves, as revealed by a decrease in CO_2_ assimilation, chlorophyll *a* fluorescence, and the repression of photosynthesis-related genes *psbP1*, *rbcL, rbcS, SBP* [17,23], *PRK*, and *Lhca3* [17] probably due to a lower activity of Rubsico or carbonic anhydrase [18]. Other metabolic alterations detected in asymptomatic leaves of esca-affected vines include a slight upregulation of the aquaporin-encoding water-stress-related gene *TIP1* [23]. In this study, resistance mechanisms activated in asymptomatic leaves seem to first involve the use of conserved antimicrobial compounds by the vines to respond to infection rapidly, as revealed by the decreased levels of phenolic compounds (Figure 1 and Figure 3).

It is known that esca-associated fungi require several years of wood colonization to establish infection in the leaves [19]. Moreover, ”Esca complex” is characterized by partial remission or total disappearance of foliar symptoms on plants in some years [12]. The year-to-year fluctuation in symptom expression has been attributed to the combination of optimal circumstances which include rainfall [5], and the occurrence of abiotic stresses such as drought [20]. It has also been hypothesized that each season, the newly formed vessels redefining the vascular system of the vine may affect the appearance of foliar symptoms [27]. Another hypothesis is that local defense reaction is the consequence of the development of drying zones and discolorations after a sudden sap disruption after or along the apparition and development of leaf stripe symptoms [27]. A complementary hypothesis could be that symptoms sometimes do not appear because of the activation of constitutive defenses. These pre-formed compounds could be effective in restraining the propagation of fungi in the wood and the translocation of their metabolites to the leaves, depending on the years and environmental conditions. The induced production of phenolic compounds would only occur after the metabolites have reached the leaves at doses sufficient to incite symptoms. This assumption is strengthened by the finding that ex vivo, stilbenes do not influence the damaging effects of *Pa. chlamydospora* on healthy leaves [26], which indicates that phytoalexins are synthesized in the leaves not before, but after, the apparition GLSD symptoms.

## 4. Materials and Methods

### 4.1. Site Characterization: Cultivar, Location, and Weather

Experiments were performed on *V. vinifera* L. ‘Malvasia’ composed of 21–24-year-old plants in the vineyard of Quinta de Nossa Senhora de Loures (465 m, 41° 17.12’ 31’’ N, 7° 44.07’ 22’’ W) in Vila Real, Portugal. The vineyard has 1247 vines grafted on 196-17-Castel rootstock and trained to a bilateral cordon according to the royal-type trellis system, on an area of 0.27 ha. The vines were planted at a distance of 1.80 × 1.20 m in 22 longitudinal rows. The climatic conditions in the Quinta are characterized by an average annual air temperature of 14.35 °C (2.04/29.23 °C day/night) and 814 mm annual precipitation, with 75% relative humidity and a 16-h photoperiod (1350 μmol·m^−2^·s^−1^). The vineyard is located on Anthrosol (62% sand, 25% silt, 13% clay; pH 4.2) and the vines are managed without irrigation. Pruning, fertilization, and plant protection practices are undertaken annually according to local practices. The position of the vineyard in the Quinta allows all vines to be grown under the same soil and climatic conditions. The vineyard is naturally infected with esca-associated fungi, and since 2010, research at the experimental field focused on the prevention and cure of “Esca complex.”

### 4.2. Sampling Procedure

A characteristic trait of “Esca complex” is the unpredictable year-to-year discontinuity in foliar symptomatic expression [5,15,19,25]. Thus, to assess the incidence of the affliction, vines were inspected over four years through visual observations of leaf and berry symptoms and internal observations of wood symptoms by destructive means.

Several vines that did not show external symptoms since 2010 when work started at the experimental site were inspected during a four-year study period for the presence of discolorations associated with “Esca complex;” these vines were characterized as “apparently healthy” by several authors [5,11,13,14,17,23,26,28]. In this study, it was decided that an internal inspection of the wood was necessary before selecting “apparently healthy” vines. Therefore, wood cores were retrieved with a sterilized Pressler increment borer at 30 and 110 cm above the ground from the trunk of the vines, as described in [19]. Based on the analysis of wood cores, the vines were categorized into two groups. The first group consisted of vines that did not exhibit symptoms either in the trunk or in the leaves; these vines were presumed healthy and considered as “controls,” as suggested in several papers [7,10,19,25]. Woods cores were subsequently subjected to fungal isolation and identification as described in [6]; *Pa. chlamydospora*, *Pm. minimun*, and *F. mediterranea* were usually not identified in these wood cores. The second group consisted of vines with brown necrosis and dark streaking of the xylem vessels, or BWS vines. These vines did not exhibit visible leaf or berry symptoms during the four-year survey. *Phaeomoniella chlamydospora* and *Pm. minimun* were identified in these wood cores, along with some *Phaeoacremonium*, *Botryospaeriaceae*, and other species (data not shown). The wood deterioration characteristic of “white rot” was not observed. GLSD was the prevalent form of “Esca complex” in the vineyard. Some GLSD vines had both symptomatic and asymptomatic shoots (one cordon symptomatic and one cordon asymptomatic), and they were selected for the study; such vines were also studied by several authors [4,14,17,24,28]. Other vines that showed GLSD leaf symptoms in a one or more inspection years and in some years only berry symptoms were also studied; however, this was a rare observation in the vineyard.

GLSD leaf symptoms at different degrees of severity were easily identifiable in the field. Leaf symptoms appeared between late June and early August, and although they usually increased in severity with plant growth, this increase was highly variable. In order to understand the biosynthesis of phenolic compounds by symptomatic expression, rather than selecting leaves with different degrees of symptom severity, vines with the majority of their leaves showing the same degree of symptom severity at harvest were targeted. In some vines, small chloroses characteristic of GLSD appeared, but did not evolve rapidly into spotting/scorching or tiger stripes. At the time of berry harvest, the surface of most leaves on these vines was still covered with discolorations, although some leaves started producing spotting/scorching or assuming the “tiger stripes” pattern (GLSD severity stage 1). At harvest, GLSD symptoms appeared in some vines as mainly chlorotic/spotting/scorching zones scattered over the leaf lamina (GLSD severity stage 2) or mainly tiger striped leaves (GLSD severity stage 3). An attempt was made to group vines exhibiting apoplectic symptoms; these symptoms appeared in a highly discontinuous manner in time (usually between early August and early September) and space in the vineyard. All selected vines were numbered and marked according to their place in the lines and rows.

### 4.3. Sample Collection

**Figure 6:**
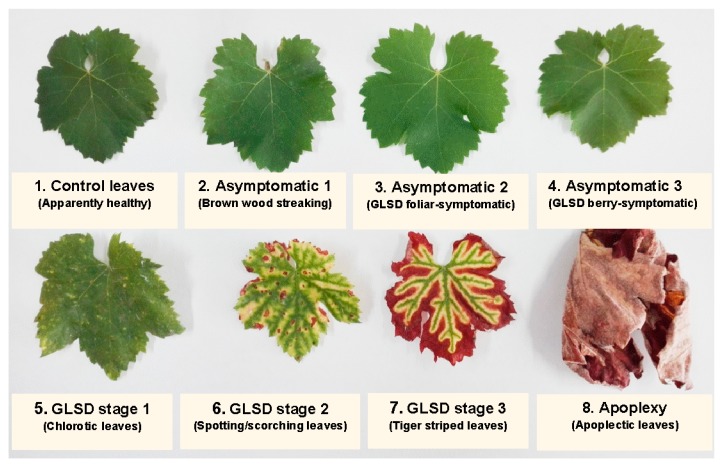
Description of the sampling procedure: A view of the foliar morphology of asymptomatic and symptomatic leaves of *Vitis vinifera* L. ‘Malvasia’ affected by brown wood streaking, grapevine leaf stripe (GLSD) and apoplexy.

The occurrence of symptoms in the vineyard allowed the collection of different sets of leaves, which were divided into eight groups (Figure 6): (1) Asymptomatic leaves from apparently healthy vines (control); (2) asymptomatic leaves from BWS vines (asymptomatic 1), to analyze the systemic effects of trunk-localized fungi attack; (3) asymptomatic leaves from asymptomatic cordons on GLSD vines (asymptomatic 2), to assess whether the biosynthesis of defensive compounds was similar in symptomatic and asymptomatic parts of the same vine; (4) asymptomatic leaves from GLSD vines with berry symptoms (asymptomatic 3), to analyze the systemic effects of berry-localized infection; (5) symptomatic leaves from vines with initial symptoms of GLSD i.e., chlorotic leaves (GLSD stage 1); (6) symptomatic leaves from vines with moderate symptoms of GLSD i.e., chlorotic/spotting/scorching leaves (GLSD stage 2); (7) symptomatic leaves from vines with advanced symptoms of GLSD i.e., tiger striped leaves (GLSD stage 3); (8) symptomatic leaves from apoplectic vines (apoplexy). In the field, apoplexy appeared quickly, affecting the entire vine with total wilt and immediate drying caused by the hot weather (an average of 32 °C day temperature during apoplexy expression); thus, apoplectic leaves were harvested and studied already dried as in [23]. All symptomatic leaves were collected to study locally induced defenses.

All samples were collected mid-September, one day prior to berry harvesting. This ensured that the leaves were at the same stage of maturity. For each leaf-group, four vines were used for sampling and were considered as replicates. Six to twelve leaves of the same size from different parts of a vine were selected. Only two vines exhibited berry symptoms at harvest; hence, two sets of leaves were harvested from each vine to make four replicates, allowing for statistical comparisons. Leaves were immediately frozen in the field with liquid nitrogen to halt enzymatic activities and stored at −80 °C. Prior to use, the leaves were lyophilized, finely powdered with a hand blender, and sieved (0.2-mm mesh).

### 4.4. Determination of Total Amounts of Phenolic Compounds

Phenolic compounds were extracted using an optimized laboratory protocol. After defatting with 1 mL hexane for 16 h, 0.2 g samples were extracted using 1 mL 70% methanol added with 10 μL naringin as an internal standard, during ultrasonication in ice water for 20 min. The extract was centrifuged at 13,000× *g* for 15 min (25 °C), and the extraction was repeated using the pellet. The combined supernatants were pre-purified on a Sep-Pak C18 cartridge (Waters, Milford, MA, USA) to remove chlorophylls, and then filtered through a Spartan 13/0.2 RC filter (Whatman, Dassel, Germany). The filtrate was used for the determination of total phenolic content (TPC) in mg gallic acid equivalent [GAE]·g^−1^ using the Folin–Ciocalteu method as described in [36]; total flavonoid content (TFC) in mg catechin equivalent [CAE]·g^−1^ using aluminum chloride as described in [36]; and total proanthocyanidin content (TPAC) in mg [GAE]·g^−1^ using polyvinylpyrrolidone, as described in [37]. The total anthocyanin content (TAC) was estimated using the pH differential assay [38], and the results were expressed in mg cyanidin 3-*O*-glucoside [CGE]·g^−1^.

### 4.5. Chromatographic Separation and Identification of Phenolic Compounds

The quantitative analysis of individual phenolic compounds was carried out on a Gilson (Villers-le-bel, France) high-performance liquid chromatography (HPLC) instrument consisting of an autosampler, binary pump, column compartment, and a Finnigan photodiode array detector (DAD 81401; Thermo Electron, San Jose, CA, USA). Chromatography was performed on 10 μL samples of the phenolic filtrate injected into the HPLC onto a C18 column (5 μm, 250 × 4.5 mm i.d.) supplied from Sigma/Aldrich (Steinheim, Germany), and maintained at 25 °C. The solvent system consisted of 0.1% trifluoroacetic acid in water (mobile phase A) and 0.1% trifluoroacetic acid in acetonitrile (mobile phase B). Elution was performed at a constant flow rate of 1 mL.min^−1^ using a linear gradient program starting with 100% mobile phase A for 5 min, decreasing to 80% at 15 min, 50% at 30 min, 0% at 45 min, and then reverting to 100% at 55 until reaching 60 min.

The detection of compounds by DAD was conducted by scanning between 210–520 nm, with a resolution of 1.2 nm. Eluting peaks were monitored at 280, 320, 360, and 520 nm for hydroxybenzoic acids and other low molecular weight compounds, hydroxycinnamic acids and stilbenes, flavonoids, and anthocyanins, respectively, using the software Excalibur 2.0, which generated a three-dimensional dataset (absorbance, retention time, and wavelength). Eluting peaks at 450 nm were also monitored because two peaks were consistently observed with large areas at that wavelength. The peaks were selected using both the Gensis and the ICIS detection algorithms of Xcalibur. The threshold for quantification by peak areas was 5000 μAU·min^−1^, and compounds whose peak areas were below this value were considered “non-detected.”

For identification, 38 reference compounds previously reported in grapevine leaves [10,12,13,19,22,25,26,33], and representatives of the chemical classes under study were purchased (Table S1); they were also separated by HPLC. Peaks were identified with “some certainty” to compounds by matching UV/vis spectra and retention times with those of the reference compounds. The remaining peaks were putatively identified by comparison with UV/vis bibliographic data. Some peaks could not match to any compounds or phenolic group and were labeled as “unknown.” Compounds were quantified by dividing their peak areas with that of the internal standard (naringin) and the results were converted to mg·kg^−1^ after correction by the peak area of the reference, its response factor, and the amount of biomass extracted. For compounds identified putatively, quantification was carried out using reference compounds with similar chemical characteristics as shown in Table S1.

### 4.6. Extraction, Separation, and Identification of Fatty Acids

The extraction of lipids was based on the method presented in [39]. Leaf samples (5 mg) were added with 0.8 mL water and 2 mL methanol in a DSR-2800V rotary shaker (Digisystem Laboratory Instruments Inc, Taipei, Taiwan) at room temperature; after continuous shaking for 5 min, 1 mL chloroform was added, and it was followed by agitation for 5 min. The mixture was centrifuged for 5 min at 2000× *g* (25 °C). The supernatant was collected and 2 mL chloroform/water (1/1, *v*/*v*) and five drops of 100 mM KCl were added. After vortexing, the mixture was centrifuged for 5 min at 2000× *g* (25 °C). The lipid fraction in the bottom layer was collected and the chloroform phase was evaporated to dryness under nitrogen. The dried extract was then transesterified with 5 mL 14% boron trifluoride in methanol under nitrogen at 70 °C for 60 min. Transesterified lipids were extracted by adding 5 mL hexane, followed by 3 min of vortexing. The upper phase, constituting fatty acid methyl esters (FAME), was collected and 1 g Na_2_SO_4_ was added to remove water.

FAME were separated via capillary gas chromatography (CG) using Shimadzu GC-2010 Plus (Shimadzu, Kyoto, Japan) equipped with an autosampler and an automatic split/splitless injector. Exactly 1 µL of FAME extract was injected into the GC at an inlet temperature of 270 °C and a split ratio of 5:1; compounds were separated on a 30 m long, 0.25-μm-thick-film DB-225MS column with a 0.25 mm i.d. (Agilent, Wilmington, DE, USA). The flow rate of the carrier gas (helium) was maintained at a constant value of 1 mL·min^−1^ at an inlet pressure of 200 kPa. The column temperature was maintained at 200 °C for 10 min, and it was then increased to 220 °C at a rate of 5 °C·min^−1^.

The resolved compounds were detected using a flame ionization detector (FID-2010 Plus) set at 270 °C; the compounds were identified by comparing their retention times to those of a standard FAME mixture (FAME 37, Supelco, Bellefonte, PA, USA) run under the same conditions. Quantification was achieved by integrating the peaks with the Lab Solution 5.71 software, setting the minimum peak area/height at 2000 count. The amount of each FAME was expressed as a weight percentage of the total FAMEs represented in the chromatogram.

### 4.7. Statistical analyses

All data from four replications were subjected to an analysis of variance (ANOVA) using SPSS 16.0 (SPSS Inc., Chicago, IL, USA). In the case of TPC, TPAC, TFC and TAC, each replicate was analyzed two times and the average values used in statistical analyses. The Tukey’s test was applied for assessing the mean differences, and a *P* value of ≤ 0.05 was considered as meaning statistical difference between the leaf groups.

## Supplementary Materials

The following are available online at https://www.mdpi.com/2223-7747/8/10/412/s1, Figure S1: Principal component analysis (PCA) score and loading plots of phenolic compounds in leaves of vines infected by brown wood streaking, grapevine leaf stripe and apoplexy (Esca complex), Figure S2: Principal component analysis (PCA) score and loading plots of fatty acids in leaves of vines infected by brown wood streaking, grapevine leaf stripe and apoplexy (Esca complex), Table S1: Phenolic compounds identified in *Vitis vinifera* L. cv. Malvasia leaves from healthy, brown wood streaking, grapevine leaf stripe and apoplexy-infected vines and listed in decreasing order based on their average contents (dry weight basis), Table S2: Effect of brown wood streaking, grapevine leaf stripe and apoplexy (Esca complex) on minor phenolic compounds (average content < 1.00 mg·kg^-1^, dry weight basis) in *Vitis vinifera* L. cv. Malvasia leaves, Table S3: Fatty acids identified in *Vitis vinifera* l. cv. Malvasia leaves from healthy, brown wood streaking, grapevine leaf stripe and apoplexy-infected vines and listed n decreasing order based on their average contents (dry weight basis), Table S4: Effect of brown wood streaking, grapevine leaf stripe and apoplexy (Esca complex) on the levels (%, dry weight basis) of 24 fatty acids in *Vitis vinifera* L. cv. Malvasia leaves.
