# Supplementary material for: Exhibition of Local but Not Systemic Induced Phenolic Defenses in Vitis vinifera L. Affected by Brown Wood Streaking, Grapevine Leaf Stripe, and Apoplexy (Esca Complex)
Source: Plants (Basel). 2019 Oct 14;8(10):412. doi: 10.3390/plants8100412 (PMC6843574; doi:10.3390/plants8100412)
Supplement: Supplementary file 1 [file plants-08-00412-s001.pdf]

Exhibition of local but not systemic induced phenolic defenses in grapevine (*Vitis vinifera* L.) affected by brown wood streaking, grapevine leaf stripe, and apoplexy (Esca complex)

Piebiep Goufo, Ana C. Marques, Isabel Cortez

Supplementary Table 1: Phenolic compounds identified in *Vitis vinifera* L. cv. Malvasia leaves from healthy, brown wood streaking, grapevine leaf stripe and apoplexy-infected vines, and listed in decreasing order based on their average contents (dry weight basis), and the correlation between the content of each phenolic compound and level of esca leaf symptom severity. The disease severity is rated using a semi-quantitative scale from 1 to 4 where: 1= Grapevine Leaf Stripe Disease stage 1; 2 = Grapevine Leaf Stripe Disease stage 2; 3 = Grapevine Leaf Stripe Disease stage 3, and 4 = Apoplexy. Significant r values are highlighted in red ($P \leq 0.01$), yellow ($P \leq 0.05$), and gray ($P \leq 0.10$).

Peak number	Retention time (min)	UV spectrum (λmax)	Reference standard peak area	Response factor	Expression ($mg.kg^{-1}$ equivalent of compound)	Phenolic compound	Mode of identification	Phenolic group	Average content ($mg.kg^{-1}$)	*r* value (Pearson)	*P* value
48	23.24	358, 256, 300	2206896273	197632		quercetin-3-*O*-glucuronide	spectra, standard	flavonol	2834.43	-0.865	0.105
39	21.32	360, 265	215473412	192961	myricetin-3-*O*-glucoside	myricetin-3-*O*-galactoside	spectra	flavonol	127.68	-0.030	0.970
50	23.45	356, 254	200548040	179595		quercetin-3-*O*-glucoside	spectra, standard	flavonol	127.32	0.773	0.227
46	22.84	362, 256, 301	291706049	261229		quercetin-3-*O*-galactoside	spectra, standard	flavonol	83.35	-0.906	0.094
22	16.78	326, 298, 243	370105650	331438		caftaric acid	spectra, standard	hydroxycinnamic acid	82.78	-0.061	0.939
43	21.94	362, 298, 260	215473412	192961		myricetin-3-*O*-glucoside	spectra, standard	flavonol	60.82	-0.998	0.002
72	27.88	348, 263, 297	370709901	331979		kaempferol-3-*O*-glucoside	spectra, standard	flavonol	54.01	-0.651	0.349
24	17.56	316, 234	370105650	331438	caftaric acid	coutaric acid	spectra	hydroxycinnamic acid	49.00	-0.919	0.081
32	19.53	277, 226	20146791	180419		epicatechin	spectra, standard	hydroxyflavan	37.93	-0.791	0.209
44	22.44	353, 256	214284635	191897		quercetin-3-*O*-rutinoside	spectra, standard	flavonol	23.10	-0.476	0.524
66	26.48	354, 274	370709901	331979	kaempferol-3-*O*-glucoside	kaempferol-3-*O*-rutinoside	spectra	flavonol	13.72	0.776	0.224
31	19.29	274, 238	24096296	215788		epigallocatechin gallate	spectra, standard	hydroxyflavan	12.38	-0.840	0.160
52	23.89	362	215473412	192961	myricetin-3-*O*-glucoside	myricetin-3-*O*-rhamnoside	spectra	flavonol	9.34	-0.771	0.229
94	48.75	435, 266	24096296	215788	naringin	unknown compound 7	spectra	unknown	4.30	-0.975	0.025
37	20.73	278, 240	46170855	413470		epicatechin gallate	spectra, standard	hydroxyflavan	3.70	-0.867	0.133
13	15.03	274, 212, 235	30911159	276816		epigallocatechin	spectra, standard	hydroxyflavan	3.40	-0.940	0.050
68	27.05	348, 265	370709901	331979	kaempferol-3-*O*-glucoside	kaempferol-3-*O*-glucuronide	spectra	flavonol	2.85	0.854	0.146
34	20.11	276, 240	30911159	276816	epigallocatechin	gallocatechin gallate	spectra	hydroxyflavan	2.52	-0.110	0.890
18	15.96	280	29132780	251109	*p*-hydroxybenzoic acid	benzoic acid derivative 5	spectra	hydroxybenzoic acid/flavan-3-ol	2.30	-0.307	0.693
62	25.86	354, 258	220689626	197632		quercetin-3-*O*-rhamnoside	spectra, standard	flavonol	2.17	0.852	0.148
65	26.38	361, 260	370709901	331979	kaempferol-3-*O*-glucoside	kaempferol-3-*O*-galactoside	spectra	flavonol	2.06	0.998	0.002
21	16.54	275, 222	14567483	130455		catechin	spectra, standard	hydroxyflavan	2.04	-0.884	0.101
53	24.12	358, 256	215473412	192961	myricetin-3-*O*-glucoside	unidentified flavonol 1	spectra	flavonol	2.02	-0.811	0.189
2	11.23	278, 214	35164451	314906		gallic acid	spectra, standard	hydroxybenzoic acid	1.87	-0.971	0.029
29	18.96	324, 296, 240	37434937	335238		caffeic acid	spectra, standard	hydroxycinnamic acid	1.86	-0.695	0.305
36	20.64	323, 289, 238	40413406	361911		ferulic acid	spectra, standard	hydroxycinnamic acid	1.83	-0.957	0.033
6	13.44	275, 322	15082206	281677		procyanidin B1	spectra, standard	hydroxyflavan	1.75	-0.793	0.207
23	17.15	270, 330	29132780	251109	*p*-hydroxybenzoic acid	benzoic acid derivative 6	spectra	hydroxybenzoic acid/flavan-3-ol	1.51	-0.271	0.729
41	21.74	353, 300, 261	215473412	192961	myricetin-3-*O*-glucoside	myricetin-3-*O*-glucuronide	spectra	flavonol	1.48	0.990	0.010
75	29.51	372, 255, 202	34672412	310499		quercetin	spectra, standard	flavonol	1.42	0.981	0.014
20	16.31	325, 286	14670043	131374	cinnamic acid	cinnamic acid derivative 7	spectra	hydroxycinnamic acid	1.41	-0.783	0.217
45	22.62	306, 318, 229	24096296	215788	naringin	unknown compound 1	spectra	unknown/stilbenoid	1.38	-0.975	0.025
3	12.74	274, 370	30911159	276816	epigallocatechin	gallocatechin	Spectra	flavan-3-ol	1.30	-0.992	0.008
26	18.15	280, 240, 370	15082206	281677	procyanidin B1	procyanidin B2	spectra	flavan-3-ol	1.26	0.475	0.685
17	15.75	280	29132780	251109	*p*-hydroxybenzoic acid	benzoic acid derivative 4	spectra	hydroxybenzoic acid /flavan-3-ol	1.07	-0.790	0.210
38	21.07	318, 238	47707554	427232		sinapic acid	spectra, standard	hydroxycinnamic acid	1.03	-0.875	0.125
64	26.17	285, 232	41560466	372183		*t*rans-resveratrol	spectra, standard	stilbenoid	0.98	-0.361	0.639
81	36.74	351, 264, 202	37070990	331979	kaempferol-3-*O*-glucoside	kaempferol-3-*O*-rhamnoside	spectra	flavonol	0.97	0.910	0.090
33	19.94	290, 326	34672412	310499	quercetin	dihydroquercetin	spectra	flavanone	0.94	-0.882	0.118
91	47.62	327	24096296	215788	naringin	unknown compound 6	spectra	unknown/stilbenoid	0.74	-0.952	0.048

95	49.04	435, 281, 232	24096296	215788	naringin	unknown compound 8	spectra	unknown	0.73	-0.978	0.022
90	47.14	358, 266	22068962	197632	quercetin-3-*O*-glucoside	unidentified flavonol 8	spectra	flavonol	0.60	.	.
78	30.75	534, 318	22918544	205241	malvidin-3-*O*-glucoside	unidentified anthocyanin 6	spectra	anthocyanin	0.60	0.057	0.943
82	37.94	366, 259	82580540	739527	isorhamnetin-3-*O*-glucoside	isorhamnetin-3-*O*-galactoside	spectra	flavonol	0.60	0.937	0.053
9	14.11	280	29132780	251109	*p*-hydroxybenzoic acid	benzoic acid derivative 1	spectra	hydroxybenzoic acid /flavan-3-ol	0.53	0.946	0.054
28	18.55	328, 305, 282	44736469	400625	caftaric acid	fertaric acid	spectra	hydroxycinnamic acid	0.51	-0.878	0.122
54	24.33	349, 254, 205	15406151	137966		luteolin-7-*O*-glucoside	spectra, standard	flavone	0.48	-0.949	0.051
59	25.27	531, 328, 283	22918544	205241	malvidin-3-*O*-glucoside	unidentified anthocyanin 1	spectra	anthocyanin	0.46	0.296	0.704
25	18.02	328, 244, 303	23908130	214103		chlorogenic acid	spectra, standard	hydroxycinnamic acid	0.46	0.271	0.729
27	18.39	260, 292	49627115	444422		vanillic acid	spectra, standard	hydroxybenzoic acid	0.40	-0.095	0.905
56	24.76	331	24096296	215788	naringin	unknown compound 2	spectra	unknown/stilbenoid	0.39	0.919	0.081
79	31.16	284, 324	34672412	310499	quercetin	hesperetin	spectra, standard	flavanone	0.36	0.970	0.030
57	24.91	324, 224	24096296	215788	naringin	unknown compound 3	spectra	unknown/stilbenoid	0.32	-0.992	0.008
4	13.17	258, 297	30415192	272375		protocatechuic acid	spectra, standard	hydroxybenzoic acid	0.29	0.111	0.889
12	14.91	338, 300	14670043	131374	cinnamic acid	cinnamic acid derivative 3	spectra	hydroxycinnamic acid	0.24	-0.795	0.205
92	47.91	354	22068962	197632	quercetin-3-*O*-glucoside	unidentified flavonol 9	spectra	flavonol	0.23	-0.500	0.500
35	20.51	310, 225, 211	54049175	484022		p-coumaric acid	spectra, standard	hydroxycinnamic acid	0.22	-0.828	0.172
16	15.59	279, 247, 309	29132780	251109	*p*-hydroxybenzoic acid	benzoic acid derivative 3	spectra	hydroxybenzoic acid	0.19	0.187	0.813
76	29.93	276, 215, 203	14670043	131374		cinnamic acid	spectra, standard	cinnamic acid	0.19	0.931	0.069
77	30.32	369, 258, 390	9572329	85722		kaempferol	spectra, standard	flavonol	0.17	0.929	0.071
14	15.32	322	14670043	131374	cinnamic acid	cinnamic acid derivative 4	spectra	hydroxycinnamic acid/hydroxycoumarin	0.15	0.940	0.040
80	35.81	350	37070990	331979	kaempferol-3-*O*-glucoside	kaempferol-3-*O*-xyloside	spectra	flavonol	0.14	0.775	0.225
30	19.17	276	51251247	458966		syringic acid	spectra, standard	hydroxybenzoic acid	0.12	0.110	0.890
58	25.16	306, 318, 228	24096296	215788	naringin	unknown compound 4	spectra	unknown/stilbenoid	0.11	0.775	0.225
60	25.54	284, 230	24096296	215788	naringin	unknown compound 5	spectra	unknown/stilbenoid	0.11	-0.356	0.644
8	13.92	281, 228	29132780	251109	*p*-hydroxybenzoic acid	gentisic acid	spectra	hydroxybenzoic acid	0.10	-0.623	0.377
84	41.98	356, 256, 300	22068962	197632	quercetin-3-*O*-glucoside	unidentified flavonol 4	spectra	flavonol	0.09	0.800	0.200
87	45.52	355, 265	82580540	739527	isorhamnetin-3-*O*-glucoside	isorhamnetin-3-*O*-glucuronide	spectra	flavonol	0.08	0.248	0.752
93	48.12	356, 265, 310	22068962	197632	quercetin-3-*O*-glucoside	unidentified flavonol 10	spectra	flavonol	0.08	-0.925	0.075
51	23.81	372, 253	16551834	148225		apigenin-7-*O*-glucoside	spectra, standard	flavone	0.08	0.725	0.275
89	46.49	370	22068962	197632	quercetin-3-*O*-glucoside	unidentified flavonol 7	spectra	flavonol	0.07	-0.462	0.538
15	15.42	323, 236	14670043	131374	cinnamic acid	cinnamic acid derivative 5	spectra	hydroxycinnamic acid/hydroxycoumarin	0.07	-0.135	0.865
70	27.22	354	22068962	197632	quercetin-3-*O*-glucoside	unidentified flavonol 2	spectra	flavonol	0.07	0.836	0.164
71	27.26	355, 259, 299	22068962	197632	quercetin-3-*O*-glucoside	unidentified flavonol 3	spectra	flavonol	0.07	0.049	0.951
11	14.73	334, 288	14670043	131374	cinnamic acid	cinnamic acid derivative 2	spectra	hydroxycinnamic acid/hydroxycoumarin	0.07	0.775	0.225
69	27.12	354, 256, 267, 298	22068962	197632	quercetin-3-*O*-glucoside	unidentified flavonol 11	spectra	flavonol	0.06	0.775	0.225
88	46.02	354	82580540	739527	isorhamnetin-3-*O*-glucoside	isorhamnetin-3-*O*-rutinoside	spectra	flavonol	0.05	-0.258	0.742
19	16.23	326	14670043	131374	cinnamic acid	cinnamic acid derivative 6	spectra	hydroxycinnamic acid	0.05	0.493	0.507
73	28.71	534, 282, 313	22918544	205241	malvidin-3-*O*-glucoside	unidentified anthocyanin 4	spectra	anthocyanin	0.05	-0.868	0.132
85	42.31	362, 322	22068962	197632	quercetin-3-*O*-glucoside	unidentified flavonol 5	spectra	flavonol	0.04	0.750	0.250
10	14.39	285, 230	29132780	251109	*p*-hydroxybenzoic acid	benzoic acid derivative 2	spectra	hydroxybenzoic acid	0.04	0.526	0.474
63	26.03	532, 324	22918544	205241	malvidin-3-*O*-glucoside	unidentified anthocyanin 2	spectra	anthocyanin	0.04	-0.947	0.053
86	45.02	281, 327	22068962	197632	quercetin-3-*O*-glucoside	unidentified flavonol 6	spectra	flavonol	0.04	0.291	0.709
1	9.26	280, 308	23908130	214103	chlorogenic	quinic acid	spectra, standard	hydroxybenzoic acid	0.03	0.877	0.103
67	26.83	524, 314, 284	22918544	205241	malvidin-3-*O*-glucoside	unidentified anthocyanin 3	spectra	anthocyanin	0.03	-0.913	0.087
83	38.48	354, 265	82580540	739527		isorhamnetin-3-*O*-glucoside	spectra, standard	flavonol	0.03	0.676	0.324
40	21.56	367, 256	14670043	131374	cinnamic acid	ellagic acid	spectra, standard	hydroxydiphenic acid	0.02	0.894	0.126
7	13.8	272	29132780	251109		*p*-hydroxybenzoic acid	spectra, standard	hydroxybenzoic acid	0.02	.(a)	.
5	13.38	346, 289	14670043	131374	cinnamic acid	cinnamic acid derivative 1	spectra	hydroxycinnamic acid/hydroxycoumarin	0.02	-0.983	0.017
61	25.76	372, 253, 303, 207	30585303	273898		myricetin	spectra, standard	flavone	0.02	0.837	0.163
42	21.88	528, 348, 288	22918544	205241		malvidin-3-*O*-glucoside	spectra, standard	anthocyanin	0.02	-0.944	0.056
55	24.51	522, 344, 278	22918544	205241	malvidin-3-*O*-glucoside	malvidin-3-*O*-acetylglucoside	spectra	anthocyanin	0.02	-0.258	0.742
49	23.32	522, 280	4884682	43743		peonidin-3-*O*-acetylglucoside	spectra, standard	anthocyanin	0.01	-0.775	0.225
74	29.26	522, 312	22918544	205241	malvidin-3-*O*-glucoside	unidentified anthocyanin 5	spectra	anthocyanin	0.01	0.000	1.000
47	23	528, 270, 350	52310655	1025078		petunidin-3-*O*-acetylglucoside	spectra, standard	anthocyanin	0.00	.	.

Supplementary Table 2: Effect of brown wood streaking, grapevine leaf stripe and apoplexy (Esca complex) on minor phenolic compounds (average content < 1.00 $mg.kg^{-1}$, dry weight basis) in *Vitis vinifera* L. cv. Malvasia leaves. Data are presented as mean ± SD of four replicates. Row values followed by different letters are statistically different at $P \leq 0.05$ (ANOVA, Tukey's test). GLSD = Grapevine Leaf Stripe Disease.

**Phenolic compound**	**Expression ($mg.kg^{-1}$ equivalent of compound)**	**Control leaves (Apparently healthy)**	**Asymptomatic 1 (Brown wood streaking)**	**Asymptomatic 2 (GLSD-foliar symptomatic)**	**Asymptomatic 3 (GLSD-berry symptomatic)**	**GLSD stage 1 (Chlorotic leaves)**	**GLSD stage 2 (Spotting/scorching leaves)**	**GLSD stage 3 (Tiger striped leaves)**	**Apoplexy (Apoplectic Leaves)**
**Levels decreased with increasing symptom severity**									
unknown compound 3	naringin	0.15 ± 0.01 b	0.25 ± 0.02 b	0.42 ± 0.02 ab	0.28 ± 0.01 b	0.60 ± 0.06 a	0.46 ± 0.02 ab	0.24 ± 0.01 b	0.13 ± 0.02 b
unknown compound 5	naringin	0.05 ± 0.01 ef	0.01 ± 0.01 f	0.21 ± 0.02 ab	0.03 ± 0.00 f	0.23 ± 0.05 a	0.09 ± 0.00 de	0.14 ± 0.02 cd	0.16 ± 0.03 be
luteolin-7-*O*-glucoside		0.02 ± 0.00 d	0.03 ± 0.01 d	0.03 ± 0.00 d	0.00 ± 0.00 d	1.28 ± 0.06 a	1.27 ± 0.03 a	0.81 ± 0.02 b	0.39 ± 0.03 c
unknown compound 8	naringin	1.12 ± 0.03 a	1.18 ± 0.02 a	0.90 ± 0.05 ab	1.13 ± 1.13 a	0.73 ± 0.01 b	0.54 ± 0.03 c	0.16 ± 0.02 d	0.06 ± 0.02 d
unidentified anthocyanin 2	malvidin-3-*O*-glucoside	0.08 ± 0.02 a	0.05 ± 0.01 ab	0.09 ± 0.01 a	0.06 ± 0.01 ab	0.03 ± 0.03 bc	0.02 ± 0.03 bc	0.00 ± 0.00 c	0.00 ± 0.00 c
unidentified anthocyanin 3	malvidin-3-*O*-glucoside	0.07 ± 0.02 a	0.02 ± 0.00 abc	0.06 ± 0.01 a	0.03 ± 0.00 ab	0.03 ± 0.00 ab	0.01 ± 0.00 c	0.00 ± 0.00 d	0.00 ± 0.00 d
isorhamnetin-3-*O*-rutinoside	isorhamnetin-3-*O*-glucoside	0.05 ± 0.01 b	0.07 ± 0.01 b	0.06 ± 0.01 b	0.18 ± 0.04 a	0.00 ± 0.00 c	0.05 ± 0.01 bc	0.00 ± 0.00 c	0.00 ± 0.00 c
malvidin-3-*O*-glucoside		0.01 ± 0.13 ab	0.02 ± 0.00 a	0.00 ± 0.00 a	0.02 ± 0.00 a	0.02 ± 0.00 a	0.02 ± 0.00 a	0.01 ± 0.00 ab	0.00 ± 0.03 b
gentisic acid	*p*-hydroxybenzoic acid	0.11 ± 0.00 b	0.15 ± 0.02 b	0.13 ± 0.01 b	0.03 ± 0.04 c	0.12 ± 0.00 b	0.28 ± 0.03 a	0.00 ± 0.00 c	0.00 ± 0.00 c
p-coumaric acid		0.38 ± 1.06 a	0.35 ± 0.04 a	0.24 ± 0.01 ab	0.12 ± 0.01 b	0.36 ± 0.02 a	0.13 ± 0.01 b	0.09 ± 0.01 b	0.10 ± 0.02 b
unidentified flavonol 9	quercetin-3-*O*-glucoside	0.22 ± 0.01 c	0.26 ± 0.02 c	0.20 ± 0.03 c	0.58 ± 0.06 a	0.13 ± 0.02 c	0.39 ± 0.02 b	0.00 ± 0.00 c	0.03 ± 0.00 c
fertaric acid	caftaric acid	0.19 ± 0.01 d	0.13 ± 0.01 d	0.10 ± 0.05 d	1.02 ± 0.05 b	1.69 ± 0.12 a	0.45 ± 0.02 c	0.39 ± 0.06 c	0.13 ± 0.10 d
dihydroquercetin	quercetin	2.59 ± 0.03 a	0.96 ± 0.06 c	0.02 ± 0.00 d	0.02 ± 0.01 d	1.59 ± 0.10 b	0.96 ± 0.03 c	1.16 ± 0.04 c	0.18 ± 0.04 d
cinnamic acid derivative 1	cinnamic acid	0.00 ± 0.01 b	0.01 ± 0.01 b	0.01 ± 0.01 b	0.04 ± 0.00 a	0.04 ± 0.00 ab	0.03 ± 0.00 ab	0.02 ± 0.00 ab	0.00 ± 0.00 b
unknown compound 6	naringin	0.28 ± 0.01 d	0.63 ± 0.03 c	0.27 ± 0.02 d	2.23 ± 0.24 a	1.13 ± 0.08 b	0.95 ± 0.03 b	0.27 ± 0.04 d	0.19 ± 0.11 d
unidentified flavonol 10	quercetin-3-*O*-glucoside	0.00 ± 0.00 c	0.00 ± 0.00 c	0.00 ± 0.00 c	0.33 ± 0.02 a	0.13 ± 0.01 b	0.13 ± 0.03 b	0.02 ± 0.02 c	0.00 ± 0.00 c
**Levels increased with increasing symptom severity**									
chlorogenic acid		0.73 ± 0.25 a	0.63 ± 0.02 a	0.56 ± 0.02 abc	0.29 ± 0.02 fg	0.12 ± 0.01 g	0.62 ± 0.06 ab	0.39 ± 0.02 cde	0.34 ± 0.01 def
unidentified anthocyanin 6	malvidin-3-*O*-glucoside	0.41 ± 0.12 b	0.50 ± 0.09 b	0.54 ± 0.12 b	0.35 ± 0.02 b	0.60 ± 0.04 ab	0.86 ± 0.01 a	0.90 ± 0.01 a	0.61 ± 0.02 ab
unidentified flavonol 2	quercetin-3-*O*-glucoside	0.05 ± 0.00 b	0.04 ± 0.01 b	0.04 ± 0.01 b	0.11 ± 0.02 ab	0.06 ± 0.02 b	0.03 ± 0.01 b	0.10 ± 0.00 ab	0.14 ± 0.01 a
unidentified anthocyanin 1	malvidin-3-*O*-glucoside	0.25 ± 0.04 ab	0.18 ± 0.01 b	0.59 ± 0.06 ab	0.33 ± 0.02 ab	0.67 ± 0.01 a	0.32 ± 0.01 ab	0.67 ± 0.02 a	0.69 ± 0.02 a
**Compounds detected only in asymptomatic leaves**									
cinnamic acid derivative 2	cinnamic acid	0.00 ± 0.03 c	0.15 ± 0.02 b	0.36 ± 0.01 a	0.00 ± 0.00 c	0.00 ± 0.00 c	0.00 ± 0.00 c	0.00 ± 0.00 c	0.01 ± 0.01 c
unknown compound 4	naringin	0.75 ± 0.00 a	0.00 ± 0.00 b	0.08 ± 0.04 b	0.07 ± 0.00 b	0.00 ± 0.00 b	0.00 ± 0.00 b	0.00 ± 0.00 b	0.01 ± 0.01 b
unidentified flavonol 8	quercetin-3-*O*-glucoside	1.56 ± 0.08 a	1.89 ± 0.23 a	1.28 ± 0.31 a	0.06 ± 0.01 ab	0.00 ± 0.00 b	0.00 ± 0.02 b	0.00 ± 0.03 b	0.00 ± 0.00 b
p-hydroxybenzoic acid		0.07 ± 0.05 a	0.03 ± 0.02 a	0.07 ± 0.01 a	0.00 ± 0.00 b	0.00 ± 0.00 b	0.00 ± 0.00 b	0.00 ± 0.00 b	0.00 ± 0.00 b
**Compounds unaffected by most symptoms**									
myricetin		0.00 ± 0.00 a	0.00 ± 0.00 a	0.02 ± 0.01 a	0.00 ± 0.00 a	0.00 ± 0.00 a	0.03 ± 0.01 a	0.05 ± 0.00 a	0.04 ± 0.01 a
apigenin-7-*O*-glucoside		0.00 ± 0.26 b	0.00 ± 0.01 b	0.22 ± 0.34 b	0.01 ± 0.01 b	0.03 ± 0.00 b	0.00 ± 0.07 b	0.00 ± 0.00 b	0.34 ± 0.12 a
unidentified flavonol 11	quercetin-3-*O*-glucoside	0.00 ± 0.20 b	0.00 ± 0.00 b	0.00 ± 0.00 b	0.02 ± 0.03 b	0.00 ± 0.00 b	0.00 ± 0.00 b	0.00 ± 0.00 b	0.49 ± 0.20 a
unidentified anthocyanin 4	malvidin-3-*O*-glucoside	0.08 ± 0.01 a	0.05 ± 0.01 ab	0.05 ± 0.01 ab	0.05 ± 0.01 ab	0.05 ± 0.00 ab	0.05 ± 0.02 ab	0.04 ± 0.01 ab	0.00 ± 0.02 b
kaempferol-3-*O*-xyloside	kaempferol-3-*O*-glucoside	0.00 ± 0.01 b	0.00 ± 0.00 b	0.00 ± 0.00 b	0.00 ± 0.00 b	0.00 ± 0.00 b	0.00 ± 0.00 b	0.00 ± 0.00 b	0.11 ± 0.01 a
vanillic acid		0.98 ± 1.09 a	0.76 ± 0.32 ab	0.90 ± 0.10 a	0.00 ± 0.07 b	0.00 ± 0.01 b	0.45 ± 0.01 ab	0.06 ± 0.08 ab	0.08 ± 0.06 ab
unidentified flavonol 3	quercetin-3-*O*-glucoside	0.03 ± 0.04 a	0.06 ± 0.09 a	0.06 ± 0.08 a	0.01 ± 0.01 a	0.08 ± 0.01 a	0.09 ± 0.01 a	0.13 ± 0.00 a	0.07 ± 0.02 a
isorhamnetin-3-*O*-glucuronide	isorhamnetin-3-*O*-glucoside	0.13 ± 0.01 b	0.21 ± 0.04 a	0.08 ± 0.01 bc	0.04 ± 0.03 bc	0.05 ± 0.01 bc	0.04 ± 0.02 bc	0.02 ± 0.02 c	0.07 ± 0.01 bc
petunidin-3-*O*-acetylglucoside		0.01 ± 0.01 a	0.00 ± 0.02 a	0.00 ± 0.00 a	0.00 ± 0.00 a	0.00 ± 0.06 a	0.00 ± 0.00 a	0.00 ± 0.03 a	0.00 ± 0.01 a
unidentified anthocyanin 5	malvidin-3-*O*-glucoside	0.04 ± 0.04 a	0.00 ± 0.00 a	0.02 ± 0.01 a	0.00 ± 0.00 a	0.01 ± 0.02 a	0.00 ± 0.01 a	0.00 ± 0.00 a	0.01 ± 0.00 a
cinnamic acid derivative 3	cinnamic acid	0.49 ± 0.02 a	0.10 ± 0.00 ab	0.36 ± 0.01 ab	0.23 ± 0.01 ab	0.26 ± 0.02 ab	0.38 ± 0.00 ab	0.11 ± 0.01 ab	0.03 ± 0.01 b
benzoic acid derivative 2	*p*-hydroxybenzoic acid	0.03 ± 0.00 bc	0.01 ± 0.00 bc	0.02 ± 0.00 c	0.01 ± 0.00 b	0.15 ± 0.00 a	0.09 ± 0.02 b	0.08 ± 0.02 bc	0.02 ± 0.00 c
peonidin-3-*O*-acetylglucoside		0.04 ± 0.41 a	0.01 ± 0.00 a	0.02 ± 0.00 a	0.01 ± 0.00 a	0.01 ± 0.00 a	0.01 ± 0.00 a	0.01 ± 0.00 a	0.00 ± 0.00 a
unidentified flavonol 7	quercetin-3-*O*-glucoside	0.06 ± 0.02 a	0.12 ± 0.04 a	0.06 ± 0.02 a	0.11 ± 0.05 a	0.04 ± 0.00 a	0.11 ± 0.06 a	0.05 ± 0.02 a	0.01 ± 0.08 a
unidentified flavonol 4	quercetin-3-*O*-glucoside	0.03 ± 0.12 a	0.04 ± 0.06 a	0.06 ± 0.01 a	0.16 ± 0.22 a	0.08 ± 0.00 a	0.10 ± 0.01 a	0.14 ± 0.01 a	0.12 ± 0.03 a
malvidin-3-*O*-acetylglucoside	malvidin-3-*O*-glucoside	0.02 ± 0.07 a	0.00 ± 0.07 a	0.02 ± 0.00 a	0.03 ± 0.01 a	0.01 ± 0.00 a	0.02 ± 0.00 a	0.01 ± 0.00 a	0.01 ± 0.00 a
cinnamic acid derivative 5	cinnamic acid	0.08 ± 0.24 a	0.06 ± 0.07 a	0.09 ± 0.01 a	0.09 ± 0.02 a	0.07 ± 0.01 a	0.06 ± 0.00 a	0.05 ± 0.00 a	0.07 ± 0.01 a
ellagic acid	cinnamic acid	0.02 ± 0.15 a	0.00 ± 0.00 a	0.00 ± 0.00 a	0.13 ± 0.04 a	0.00 ± 0.00 a	0.00 ± 0.00 a	0.02 ± 0.03 a	0.02 ± 0.03 a
protocatechuic acid		0.32 ± 0.06 a	0.19 ± 0.01 a	0.32 ± 0.16 a	0.23 ± 0.21 a	0.24 ± 0.02 a	0.37 ± 0.02 a	0.37 ± 0.01 a	0.26 ± 0.32 a
benzoic acid derivative 3	*p*-hydroxybenzoic acid	0.33 ± 0.21 a	0.26 ± 0.00 ab	0.14 ± 0.02 b	0.10 ± 0.04 b	0.13 ± 0.01 b	0.09 ± 0.02 b	0.38 ± 0.03 a	0.10 ± 0.03 b
t-resveratrol		0.92 ± 0.04 ab	0.73 ± 0.06 b	1.12 ± 0.21 ab	0.75 ± 0.01 b	0.99 ± 0.31 ab	0.67 ± 0.09 ab	2.06 ± 0.08 a	0.60 ± 0.31 b
unidentified flavonol 5	quercetin-3-*O*-glucoside	0.04 ± 0.03 a	0.04 ± 0.01 a	0.06 ± 0.01 a	0.05 ± 0.01 a	0.03 ± 0.00 a	0.04 ± 0.01 a	0.03 ± 0.01 a	0.07 ± 0.02 a
unidentified flavonol 6	quercetin-3-*O*-glucoside	0.01 ± 0.00 a	0.03 ± 0.00 a	0.04 ± 0.00 a	0.03 ± 0.01 a	0.06 ± 0.00 a	0.02 ± 0.02 a	0.07 ± 0.02 a	0.06 ± 0.02 a

Supplementary Table 3: Fatty acids identified in *Vitis vinifera* l. cv. Malvasia leaves from healthy, brown wood streaking, grapevine leaf stripe and apoplexy-infected vines, and listed in decreasing order based on their average contents (dry weight basis), and the correlation between the content of each fatty acid and level of esca leaf symptom severity. The disease severity is rated using a semi-quantitative scale from 1 to 4 where: 1= Grapevine Leaf Stripe Disease stage 1; 2 = Grapevine Leaf Stripe Disease stage 2; 3 = Grapevine Leaf Stripe Disease stage 3, and 4 = Apoplexy. Significant r values are highlighted in red ($P \leq 0.01$), yellow ($P \leq 0.05$), and gray ($P \leq 0.10$).

Peak number	Retention time (min)	Common name	Lipid number	Average content (%)	Mode of identification	*r* (Pearson correlation)	*P* (2-tailed significance)
20	32.131	γ-Linolenic acid	C18:3n6	34.65	FAME standard	-0.925	0.055
10	27.251	Palmitic acid	C16:0	15.09	FAME standard	0.658	0.342
18	31.449	Linoleic acid	C18:2n6c	5.81	FAME standard	-0.995	0.005
15	30.968	Elaidic acid	C18:1n9t	5.49	FAME standard	0.645	0.355
11	27.482	Palmitoleic acid	C16:1n7	3.71	FAME standard	0.916	0.084
22	34.167	α-Linolenic acid	C18:3n3	3.35	FAME standard	0.871	0.129
35	44.568	cis-4,7,10,13,16,19-Docosahexaenoic acid	C22:6n3	3.14	FAME standard	0.781	0.219
1	4.395	Caprylic acid	C8:0	3.05	FAME standard	0.532	0.468
19	31.662	Arachidic acid	C20:0	2.81	FAME standard	0.913	0.077
16	30.951	Oleic acid	C18:1n9c	2.22	FAME standard	-0.936	0.064
2	4.462	Capric acid	C10:0	1.86	FAME standard	0.438	0.562
28	35.828	cis-11,14,17-Eicosatrienoic acid	C20:3n3	1.68	FAME standard	0.970	0.030
7	23.667	Myristoleic acid	C14:1n5	1.64	FAME standard	0.938	0.062
21	34.022	cis-11-Eicosenoic acid	C20:1n9	1.43	FAME standard	0.882	0.118
6	23.198	Myristic acid	C14:0	1.33	FAME standard	0.493	0.507
27	35.690	Erucic	C22:1n9	1.21	FAME standard	0.979	0.021
24	34.937	cis-11,14-Eicosadienoic acid	C20:2n6	1.15	FAME standard	0.837	0.163
14	30.817	Stearic acid	C18:0	0.99	FAME standard	0.295	0.705
12	29.003	Heptadecanoic acid	C17:0	0.95	FAME standard	-0.854	0.146
3	15.237	Undecanoic acid	C11:0	0.88	FAME standard	0.815	0.185
23	34.699	Heneicosanoic acid	C21:0	0.88	FAME standard	0.842	0.158
9	25.733	cis-10-Pentadecenoic acid	C15:1n5	0.87	FAME standard	0.790	0.210
8	25.253	Pentadecanoic acid	C15:0	0.73	FAME standard	0.708	0.292
29	37.861	Arachidonic acid	C20:4n6	0.62	FAME standard	-0.789	0.211
34	44.079	Nervonic acid	C24:1n9	0.59	FAME standard	0.718	0.282
25	35.024	Behenic acid	C22:0	0.56	FAME standard	0.922	0.078
13	29.289	cis-10-Heptadecenoic acid	C17:1n7	0.52	FAME standard	0.620	0.380
31	38.973	cis-13,16-Docosadienoic acid	C22:2n6	0.50	FAME standard	0.377	0.623
4	18.328	Lauric acid	C12:0	0.48	FAME standard	0.805	0.195
33	41.140	cis-5,8,11,14,17-Eicosapentaenoic acid	C20:5n3	0.44	FAME standard	0.188	0.812
32	40.496	Lignoceric acid	C24:0	0.34	FAME standard	0.611	0.389
26	35.456	cis-8,11,14-Eicosatrienoic	C20:3n6	0.33	FAME standard	-0.775	0.225
5	20.877	Tridecanoic acid	C13:0	0.32	FAME standard	-0.069	0.931
17	31.350	Linolelaidic acid	C18:2n6t	0.23	FAME standard	-0.905	0.095
30	38.129	Tricosanoic acid	C23:0	0.15	FAME standard	-0.726	0.274
		Saturated fatty acids (SFA)		30.42		0.910	0.038
		Unsaturated fatty acids (UFA)		69.58		-0.910	0.039
		Monounsaturated fatty acids (MUFA)		17.68		0.937	0.063
		Polyunsaturated fatty acids (PUFA)		51.90		-0.944	0.046
		ω3		8.61		0.899	0.101
		ω6		43.29		-0.943	0.047
		ω5 and ω7		6.74		0.961	0.029
		ω9		10.94		0.827	0.173
		ω6/ω3		8.67		-0.823	0.177

Supplementary Table 4. Effect of brown wood streaking, grapevine leaf stripe and apoplexy (Esca complex) on the levels (%,dry weight basis) of 24 fatty acids in *Vitis vinifera* L. cv. Malvasia leaves. Data are presented as mean ± SD of four replicates. Row values followed by different letters are statistically different at $P \leq 0.05$ (ANOVA, Tukey's test). GLSD = Grapevine Leaf Stripe Disease.

Common name	Lipid number	Control leaves (Apparently healthy)	Asymptomatic 1 (Brown wood streaking)	Asymptomatic 2 (GLSD-foliar symptomatic)	Asymptomatic 3 (GLSD-berry symptomatic)	GLSD stage 1 (Chlorotic leaves)	GLSD stage 2 (Spotting/scorching leaves)	GLSD stage 3 (Tiger striped leaves)	Apoplexy (Apoplectic Leaves)
Caprylic acid	C8:0	1.30 ± 0.43 d	0.89 ± 0.27 e	1.05 ± 0.39 de	1.37 ± 0.23 d	0.83 ± 0.20 e	8.10 ± 2.03 a	4.59 ± 1.82 c	6.25 ± 0.80 b
Capric acid	C10:0	1.03 ± 0.45 c	0.63 ± 0.22 d	0.77 ± 0.36 d	0.74 ± 0.12 d	0.65 ± 0.22 d	5.00 ± 1.25 a	2.55 ± 1.19 b	3.53 ± 1.24 b
Undecanoic acid	C11:0	1.19 ± 0.08 b	0.47 ± 0.14 c	0.43 ± 0.03 c	0.58 ± 0.12 c	0.42 ± 0.08 c	1.16 ± 0.32 b	1.53 ± 0.12 a	1.31 ± 0.08 b
Lauric acid	C12:0	0.59 ± 0.14 ab	0.30 ± 0.18 bc	0.24 ± 0.07 c	0.22 ± 0.15 c	0.16 ± 0.04 c	0.75 ± 0.23 a	0.81 ± 0.25 a	0.79 ± 0.44 a
Tridecanoic acid	C13:0	0.36 ± 0.05 ab	0.40 ± 0.11 a	0.17 ± 0.05 b	0.41 ± 0.05 a	0.39 ± 0.04 ab	0.26 ± 0.11 ab	0.25 ± 0.09 ab	0.38 ± 0.06 ab
Myristic acid	C14:0	1.17 ± 0.17 bc	0.66 ± 0.08 d	0.82 ± 0.02 c	0.72 ± 0.10 cd	0.55 ± 0.06 d	2.61 ± 0.52 a	2.28 ± 0.27 ab	1.81 ± 0.11 abc
Pentadecanoic acid	C15:0	0.93 ± 0.06 ab	0.61 ± 0.20 bc	0.44 ± 0.15 c	0.61 ± 0.13 bc	0.62 ± 0.03 bc	0.73 ± 0.31 bc	1.07 ± 0.10 a	0.86 ± 0.38 ab
Heptadecanoic acid	C17:0	0.91 ± 0.26 ab	1.46 ± 0.10 a	0.88 ± 0.05 b	1.64 ± 0.40 a	1.72 ± 0.03 a	0.47 ± 0.03 c	0.28 ± 0.11 c	0.23 ± 0.17 c
Arachidic acid	C20:0	2.26 ± 0.22 c	0.07 ± 0.09 e	1.53 ± 0.26 cd	1.76 ± 0.72 cd	1.14 ± 0.56 d	4.53 ± 0.17 b	5.28 ± 1.52 ab	5.89 ± 0.39 a
Heneicosanoic acid	C21:0	0.69 ± 0.16 c	0.71 ± 0.41 c	0.77 ± 0.05 c	0.68 ± 0.15 c	0.58 ± 0.02 d	1.11 ± 0.71 b	1.32 ± 0.20 a	1.23 ± 0.16 ab
Behenic acid	C22:0	0.74 ± 0.32 b	0.23 ± 0.03 c	0.26 ± 0.06 c	0.31 ± 0.11 c	0.17 ± 0.05 c	0.66 ± 0.30 b	1.06 ± 0.37 a	1.02 ± 0.19 a
Tricosanoic acid	C23:0	0.27 ± 0.09 ab	0.37 ± 0.03 a	0.01 ± 0.09 d	0.22 ± 0.08 abc	0.16 ± 0.03 bcd	0.11 ± 0.12 bcd	0.00 ± 0.00 d	0.07 ± 0.03 d
Lignoceric acid	C24:0	0.40 ± 0.04 b	0.32 ± 0.09 bc	0.20 ± 0.03 c	0.20 ± 0.04 c	0.19 ± 0.02 c	0.56 ± 0.21 a	0.33 ± 0.04 b	0.55 ±0.28 a
Myristoleic acid	C14:1n5	2.27 ± 0.66 b	0.56 ± 0.13 d	1.17 ± 0.41 cd	0.72 ± 0.49 d	0.71 ± 0.20 d	1.71 ± 0.27 bc	1.92 ± 0.24 b	4.11 ± 0.85 a
cis-10-Pentadecenoic acid	C15:1n5	0.63 ± 0.12 b	0.37 ± 0.04 c	0.46 ± 0.09 bc	0.34 ± 0.20 c	0.35 ± 0.09 c	1.58 ± 0.20 a	1.64 ± 0.66 a	1.62 ± 0.19 a
Palmitoleic acid	C16:1n7	1.87 ± 0.53 cd	2.08 ± 0.33 c	2.10 ± 0.29 c	1.68 ± 0.84 cd	1.33 ± 0.77 d	5.52 ± 0.73 b	7.47 ± 1.33 a	7.60 ± 0.17 a
cis-11-Eicosenoic acid	C20:1n9	0.80 ± 0.48 b	0.53 ± 0.10 c	0.50 ± 0.13 c	0.78 ± 0.24 b	0.47 ± 0.24 c	2.58 ± 0.83 a	2.59 ± 0.64 a	3.17 ± 0.26 a
Erucic	C22:1n9	1.10 ± 0.47 c	0.42 ± 0.10 d	0.48 ± 0.09 d	0.52 ± 0.36 d	0.25 ± 0.07 d	1.25 ± 0.16 b	2.65 ± 0.23 ab	3.02 ± 1.89 a
Nervonic acid	C24:1n9	0.52 ± 0.44 c	0.25 ± 0.14 d	0.31 ± 0.15 d	0.29 ± 0.18 d	0.14 ± 0.11 d	0.96 ± 0.45 b	1.34 ± 1.26 a	0.95 ± 0.40 b
cis-11,14-Eicosadienoic acid	C20:2n6	0.97 ± 0.34 b	0.45 ± 0.21 c	0.48 ± 0.33 c	0.78 ± 0.29 b	0.45 ± 0.12 c	2.03 ± 0.60 a	1.76 ± 0.88 a	2.33 ± 0.38 a
cis-8,11,14-Eicosatrienoic	C20:3n6	1.03 ± 0.48 a	0.30 ± 017 c	0.31 ± 0.09 c	0.77 ± 0.45 b	0.25 ± 0.11 c	0.00 ± 0.00 d	0.00 ± 0.00 d	0.00 ± 0.00 d
cis-13,16-Docosadienoic acid	C22:2n6	0.39 ± 0.18 bc	0.49 ± 0.33 b	0.32 ± 0.09 bc	0.54 ± 0.28 b	0.31 ± 0.18 bc	0.89 ± 0.62 a	0.26 ± 0.17 c	0.85 ± 0.47 a
cis-11,14,17-Eicosatrienoic acid	C20:3n3	1.85 ± 0.07 bc	1.05 ± 0.56 c	0.82 ± 0.12 d	0.65 ± 0.11 d	0.51 ± 0.31 d	2.00 ± 0.54 b	3.09 ± 0.91 a	3.47 ± 0.48 a
cis-5,8,11,14,17-Eicosapentaenoic acid	C20:5n3	0.78 ± 0.19 a	0.24 ± 0.11 b	0.30 ± 0.11 b	0.19 ± 0.07 b	0.18 ± 0.10 b	0.81 ± 0.19 a	0.65 ± 0.10 a	0.37 ± 0.43 b
Saturated fatty acids (SFA)		25.38 ± 1.23 d	22.04 ± 1.69 e	25.68 ± 0.85 d	26.10 ± 2.53 d	22.45 ± 0.52 e	37.53 ± 0.85 c	40.82 ± 2.24 b	43.37 ± 2.37 a
Unsaturated fatty acids (UFA)		74.62 ± 1.23 b	77.96 ± 1.69 a	74.33 ± 0.85 b	73.90 ± 2.53 b	77.55 ± 0.52 a	62.48 ± 0.85 c	59.19 ± 2.24 d	56.63 ± 2.37 e
Monounsaturated fatty acids (MUFA)		15.19 ± 1.78 c	9.03 ± 1.52 e	13.65 ± 0.92 cd	12.48 ± 1.27 cd	11.02 ± 1.20 de	18.73 ± 0.51 b	31.10 ± 3.29 a	30.25 ± 2.66 a
Polyunsaturated fatty acids (PUFA)		59.43 ± 2.04 b	68.94 ± 3.01 a	60.67 ± 1.71 b	61.42 ± 2.48 b	66.53 ± 0.98 a	43.75 ± 0.99 c	28.09 ± 1.29 d	26.38 ± 2.52 d
ω3		6.36 ± 1.97 d	4.13 ± 0.43 e	9.44 ± 1.23 c	3.99 ± 0.86 e	3.32 ± 0.65 e	12.75 ± 1.60 b	12.83 ± 1.04 b	16.05 ± 2.73 a
ω6		53.07 ± 3.24 c	64.81 ± 3.22 a	51.24 ± 1.44 c	57.43 ± 2.20 b	63.22 ± 1.50 a	31.00 ± 1.94 d	15.26 ± 0.97 e	10.33 ± 0.57 f
ω5 and ω7		5.18 ± 1.08 d	3.45 ± 0.34 ef	4.43 ± 0.73 de	3.11 ± 0.64 ef	2.79 ± 0.82 f	9.25 ± 0.90 c	11.81 ± 1.12 b	13.90 ± 1.22 a
ω9		10.01 ± 0.93 c	5.58 ± 1.19 d	9.22 ± 0.22 c	9.37 ± 0.63 c	8.23 ± 1.03 c	9.48 ± 0.60 c	19.28 ± 3.75 a	16.35 ± 1.97 b
ω6/ω3		9.18 ± 3.57 c	15.88 ± 2.19 b	5.49 ± 0.70 d	14.82 ± 2.62 b	19.68 ± 4.23 a	2.47 ± 0.46 de	1.20 ± 0.13 e	0.66 ± 0.13 e

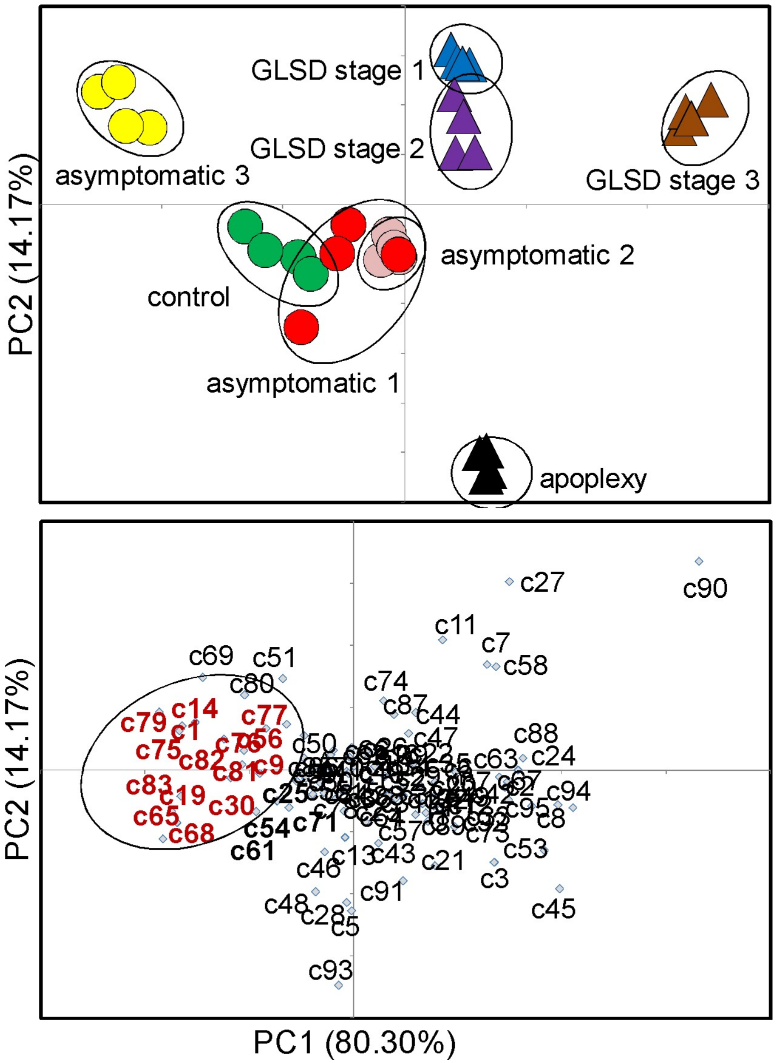

PC1+		PC1-		PC2+		PC2-	
Peak no	Value	Peak no	Value	Peak no	Value	Peak no	Value
c90	0.33	c64	-0.01	c90	0.34	c18	-0.01
c45	0.20	c18	-0.01	c27	0.30	c59	-0.01
c94	0.20	c4	-0.01	c11	0.21	c82	-0.01
c27	0.18	c5	-0.01	c7	0.17	c29	-0.01
c53	0.17	c13	-0.01	c58	0.17	c24	-0.01
c8	0.16	c23	-0.01	c69	0.15	c54	-0.01
c63	0.16	c55	-0.01	c51	0.15	c86	-0.01
c88	0.15	c93	-0.01	c80	0.12	c52	-0.02
c95	0.15	c36	-0.02	c74	0.11	c60	-0.02
c2	0.15	c66	-0.02	c47	0.09	c37	-0.02
c67	0.14	c62	-0.02	c87	0.09	c2	-0.02
c24	0.14	c46	-0.03	c44	0.09	c78	-0.02
c33	0.14	c15	-0.03	c14	0.08	c20	-0.02
c73	0.13	c78	-0.03	c56	0.07	c67	-0.03
c42	0.13	c48	-0.04	c1	0.07	c64	-0.03
c58	0.10	c70	-0.04	c76	0.07	c55	-0.03
c3	0.10	c84	-0.04	c79	0.06	c15	-0.04
c7	0.09	c41	-0.05	c75	0.06	c84	-0.04
c6	0.08	c86	-0.05	c77	0.06	c32	-0.04
c92	0.08	c50	-0.05	c61	0.05	c38	-0.04
c11	0.08	c59	-0.05	c26	0.04	c31	-0.04
c12	0.08	c60	-0.05	c66	0.03	c83	-0.04
c21	0.08	c77	-0.05	c81	0.03	c49	-0.04
c37	0.08	c30	-0.06	c72	0.03	c36	-0.05
c49	0.07	c56	-0.06	c39	0.03	c10	-0.05
c89	0.07	c51	-0.07	c22	0.03	c95	-0.05
c17	0.06	c25	-0.07	c16	0.03	c25	-0.05
c20	0.06	c69	-0.08	c62	0.02	c17	-0.05
c87	0.06	c80	-0.09	c85	0.02	c12	-0.06
c35	0.05	c71	-0.09	c50	0.02	c94	-0.06
c47	0.05	c82	-0.11	c63	0.02	c8	-0.06
c31	0.05	c81	-0.11	c35	0.01	c30	-0.06
c91	0.05	c9	-0.11	c9	0.01	c92	-0.07
c38	0.04	c54	-0.12	c40	0.01	c23	-0.07
c10	0.04	c61	-0.13	c34	0.01	c65	-0.07
c44	0.03	c76	-0.15	c70	0.01	c71	-0.07
c32	0.03	c14	-0.15	c88	0.00	c89	-0.07
c29	0.03	c1	-0.17	c41	0.00	c42	-0.08
c74	0.03	c83	-0.17	c4	0.00	c33	-0.08
c22	0.02	c79	-0.17	c6	0.00	c19	-0.08
c43	0.02	c19	-0.17			c73	-0.08
c39	0.02	c65	-0.18			c68	-0.09
c57	0.02	c68	-0.18			c53	-0.11
c34	0.01	c75	-0.19			c13	-0.11
c52	0.01					c43	-0.12
c72	0.01					c57	-0.13
c16	0.01					c46	-0.13
c40	0.00					c3	-0.15
c26	0.00					c21	-0.15
c28	0.00					c91	-0.18
c85	0.00					c45	-0.19
						c48	-0.20
						c5	-0.21
						c28	-0.23
						c93	-0.35

Supplementary Figure 1. Principal component analysis (PCA) score and loading plots of phenolic compounds in leaves of vines infected by brown wood streaking, grapevine leaf stripe and apoplexy (Esca complex). Highlighted in red are 15 compounds whose levels progressively increased with increasing foliar symptom severity. C75 = quercetin, C14 = cinnamic acid derivative 4, C56 = unknown compound 2, C1 = quinic acid, C76 = cinnamic acid, C79 = hesperetin, C30 = syringing acid, C19 = cinnamic acid derivative 6, C9 = benzoic acid derivative 1, C82 = isorhamnetin-3-*O*- galactoside, C83 = isorhamnetin-3-*O*- glucoside, C77 = kaempferol, C81 = kaempferol-3-*O*-rhamnoside, C68 = kaempferol-3-*O*-glucuronide, C65 = kaempferol-3-*O*-galactoside. PC1 and PC2 eigenvalues for phenolic compounds discriminated by PCA are also shown in a table, and in colour in the table are compounds contributing the most to the separation of leaf groups. Data were square-root transformed prior to PCA, which was performed on unit variance auto-scale using an Excel statistical tool (http://prime.psc.riken.jp/Metabolomics_Software/StatisticalAnalysisOnMicrosoftExcel/).

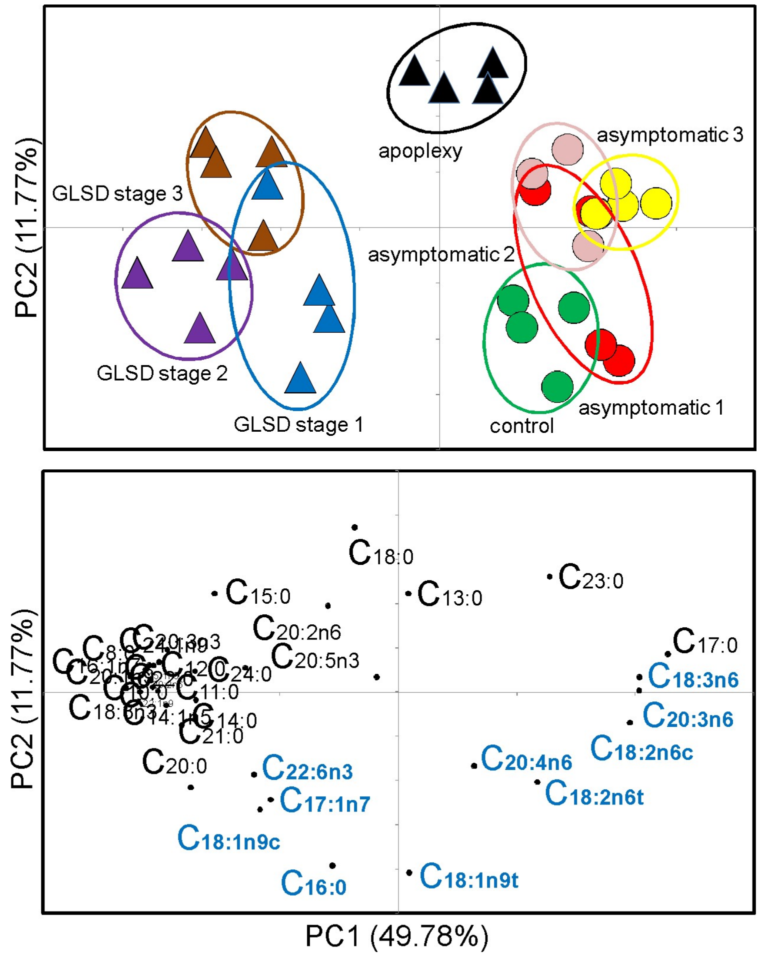

PC1+		PC1-		PC2+		PC2-	
Peak no	Value	Peak no	Value	Peak no	Value	Peak no	Value
C18:3n6	0.23	C20:5n3	-0.04	C18:0	0.37	C14:0	-0.02
C17:0	0.20	C16:0	-0.06	C23:0	0.26	C14:1n5	-0.03
C20:3n6	0.20	C22:2n6	-0.06	C24:0	0.22	C21:0	-0.07
C23:0	0.18	C17:1n7	-0.11	C13:0	0.22	C18:2n6c	-0.08
C18:2n6c	0.13	C18:1n9c	-0.12	C22:2n6	0.20	C20:4n6	-0.17
C18:2n6t	0.12	C22:6n3	-0.12	C12:0	0.10	C22:6n3	-0.19
C20:4n6	0.06	C15:0	-0.13	C18:3n6	0.09	C18:2n6t	-0.20
C18:1n9t	0.01	C24:0	-0.16	C10:0	0.07	C20:0	-0.22
C13:0	0.01	C14:0	-0.17	C20:2n6	0.06	C17:1n7	-0.24
C18:0	-0.02	C24:1n9	-0.17	C20:3n3	0.06	C18:1n9c	-0.26
		C20:0	-0.18	C15:0	0.06	C16:0	-0.39
		C21:0	-0.18	C8:0	0.05	C18:1n9t	-0.41
		C12:0	-0.20	C24:1n9	0.05		
		C14:1n5	-0.20	C20:1n9	0.04		
		C10:0	-0.20	C15:1n5	0.04		
		C22:1n9	-0.20	C17:0	0.03		
		C20:3n3	-0.21	C20:5n3	0.03		
		C22:0	-0.21	C11:0	0.03		
		C11:0	-0.21	C22:0	0.01		
		C20:2n6	-0.21	C18:3n3	0.01		
		C8:0	-0.21	C20:3n6	0.00		
		C16:1n7	-0.22	C22:1n9	0.00		
		C20:1n9	-0.23	C16:1n7	0.00		
		C15:1n5	-0.23				
		C18:3n3	-0.23				

Supplementary Figure 2. Principal component analysis (PCA) score and loading plots of fatty acids in leaves of vines infected by brown wood streaking, grapevine leaf stripe and apoplexy (Esca complex). Highlighted in blue are fatty acids whose levels increased in some asymptomatic leaves. PC1 and PC2 eigenvalues for fatty acids discriminated by PCA are also shown in a table, and in colour in the table are compounds contributing the most to the separation of leaf groups. Data were square-root transformed prior to PCA, which was performed on unit variance auto-scale using an Excel statistical tool (http://prime.psc.riken.jp/Metabolomics_Software/StatisticalAnalysisOnMicrosoftExcel/).
